# Clonal Architectures and Driver Mutations in Metastatic Melanomas

**DOI:** 10.1371/journal.pone.0111153

**Published:** 2014-11-13

**Authors:** Li Ding, Minjung Kim, Krishna L. Kanchi, Nathan D. Dees, Charles Lu, Malachi Griffith, David Fenstermacher, Hyeran Sung, Christopher A. Miller, Brian Goetz, Michael C. Wendl, Obi Griffith, Lynn A. Cornelius, Gerald P. Linette, Joshua F. McMichael, Vernon K. Sondak, Ryan C. Fields, Timothy J. Ley, James J. Mulé, Richard K. Wilson, Jeffrey S. Weber

**Affiliations:** 1 The Genome Institute, Washington University in St. Louis, St. Louis, Missouri, United States of America; 2 Department of Medicine, Washington University in St. Louis, St. Louis, Missouri, United States of America; 3 Department of Genetics, Washington University in St. Louis, St. Louis, Missouri, United States of America; 4 Siteman Cancer Center, Washington University in St. Louis, St. Louis, Missouri, United States of America; 5 Donald A. Adam Comprehensive Melanoma Research Center, Moffitt Cancer Center, Tampa, Florida, United States of America; 6 Department of Surgery, Washington University in St. Louis, St. Louis, Missouri, United States of America; Duke-National University of Singapore Graduate Medical School, Singapore

## Abstract

To reveal the clonal architecture of melanoma and associated driver mutations, whole genome sequencing (WGS) and targeted extension sequencing were used to characterize 124 melanoma cases. Significantly mutated gene analysis using 13 WGS cases and 15 additional paired extension cases identified known melanoma genes such as *BRAF*, *NRAS*, and *CDKN2A*, as well as a novel gene *EPHA3*, previously implicated in other cancer types. Extension studies using tumors from another 96 patients discovered a large number of truncation mutations in tumor suppressors (*TP53* and *RB1*), protein phosphatases (e.g., *PTEN*, *PTPRB*, *PTPRD*, and *PTPRT*), as well as chromatin remodeling genes (e.g., *ASXL3*, *MLL2*, and *ARID2*). Deep sequencing of mutations revealed subclones in the majority of metastatic tumors from 13 WGS cases. Validated mutations from 12 out of 13 WGS patients exhibited a predominant UV signature characterized by a high frequency of C->T transitions occurring at the 3′ base of dipyrimidine sequences while one patient (MEL9) with a hypermutator phenotype lacked this signature. Strikingly, a subclonal mutation signature analysis revealed that the founding clone in MEL9 exhibited UV signature but the secondary clone did not, suggesting different mutational mechanisms for two clonal populations from the same tumor. Further analysis of four metastases from different geographic locations in 2 melanoma cases revealed phylogenetic relationships and highlighted the genetic alterations responsible for differential drug resistance among metastatic tumors. Our study suggests that clonal evaluation is crucial for understanding tumor etiology and drug resistance in melanoma.

## Introduction

The incidence of invasive melanoma in the United States in 2013 is estimated to be 76,690, with approximately 9,480 deaths [1]. While the death rate due to melanoma is relatively low, and many tumors are found at an early stage when they can be completely resected and cured, the development of metastatic disease is a harbinger of poor outcome. Though four new drugs that prolong overall or progression-free survival were recently approved for stage IV disease [2], [3], the median survival for metastatic melanoma remains poor. Work in the last decade has identified a number of common and/or driver mutations in melanoma and helped to elucidate the pathways determining melanoma oncogenesis, proliferation, and metastasis. These discoveries have led to the development of inhibitors for *BRAF* and *KIT* (C-Kit) signaling, some of which have shown benefit in melanoma patients [4], [5].

The driver mutations in *BRAF* and *NRAS* that have been identified cannot fully explain melanoma oncogenesis, as these same mutations have been found at similar rates in benign nevi, or moles [6], [7]. These common, benign skin lesions infrequently undergo malignant transformation into melanoma, but invariably remain in their growth-arrested state. *BRAF* V600E-induced oncogenic senescence has been implicated in melanoma cell cycle arrest [8], together with loss of function of genes including *TP53*, *NF2*, and *IRF1* [9], *CDKN2A* (*INK4A/ARF*) and *CDK4* [10]. In fact, loss of senescence is an important process in RAS- and RAF-induced transformation, implying that additional but still unknown genomic changes must be involved in transformation to melanoma. The mutational variants identified by WGS can provide insight into this process.

The first melanoma genome sequenced was derived from an established cell line [11] and it showed a large number of SNVs, most corresponding to a UV signature of C->T transitions [12]. Recently, several exome-based studies have been conducted to identify genes driving melanoma development. Directed sequencing has demonstrated *ERBB4* mutations in melanomas [13] and copy number analysis has revealed amplification of the histone methyltransferase gene *SETDB1* [14]. Frequent somatic mutations in *MAP3K5* and *MAP3K9* were identified in metastatic melanomas by exome sequencing [15]. A preliminary study of matched whole exome sequencing of melanomas and matched normals indicates that *GRIN2A* was frequently mutated in 14 specimens, and subsequent analysis of over 100 samples showed it to be mutated in 33% of cases [16]. *TRRAP* and *GRM3* were also found to be mutated in a small proportion of tumors, indicating that the glutamate pathway might play an important role in melanoma development and progression [17]. Another exome sequencing study described six novel melanoma genes: *PPP6C*, *RAC1*, *SNX31*, *TACC1*, *STK19*, and *ARID2* [*18*]. Recently, *PREX2*, a phosphatidylinositol-3,4,5-trisphosphate-dependent Rac exchange factor 2, was found to be mutated in 14% of 107 melanoma cases [19]. Whole genome sequencing of an acral melanoma primary and lymph nodal metastasis showed 40 SNVs in the primary, of which 39 were also present in the metastasis [20].

Our analysis of 15 metastatic melanoma tumors from 13 patients shows very high numbers of non-synonymous SNVs and it constitutes a useful catalogue of copy number alterations, insertions, deletions and translocations within those genomes. *EPHA3* (ephrin type-A receptor 3) was found to be significantly mutated in 28 melanoma cases. Extension analysis using 97 tumors from 96 patients revealed a number of truncation mutations in well-known tumor suppressors, protein phosphatases, as well as genes involved in chromatin remodeling. Notably, the majority of these truncation mutations co-occur with *BRAF* and *NRAS* mutations, suggesting a potential cooperating role during the progression of melanoma. We also performed deep sequencing of somatic mutations that uncovered the clonal structures of melanomas, helped to dissect diverse mutational mechanisms in subclones, and further established the initiation roles of *BRAF* and *NRAS* mutations in melanoma. More importantly, our comparative analysis of 4 metastases from different geographical locations in 2 cases revealed a clonal evolution path and underscored the genetic alterations responsible for drug resistance.

## Results

### Genomic analysis of the whole genome sequencing data of 15 tumors from 13 melanoma cases

**Figure 1:**
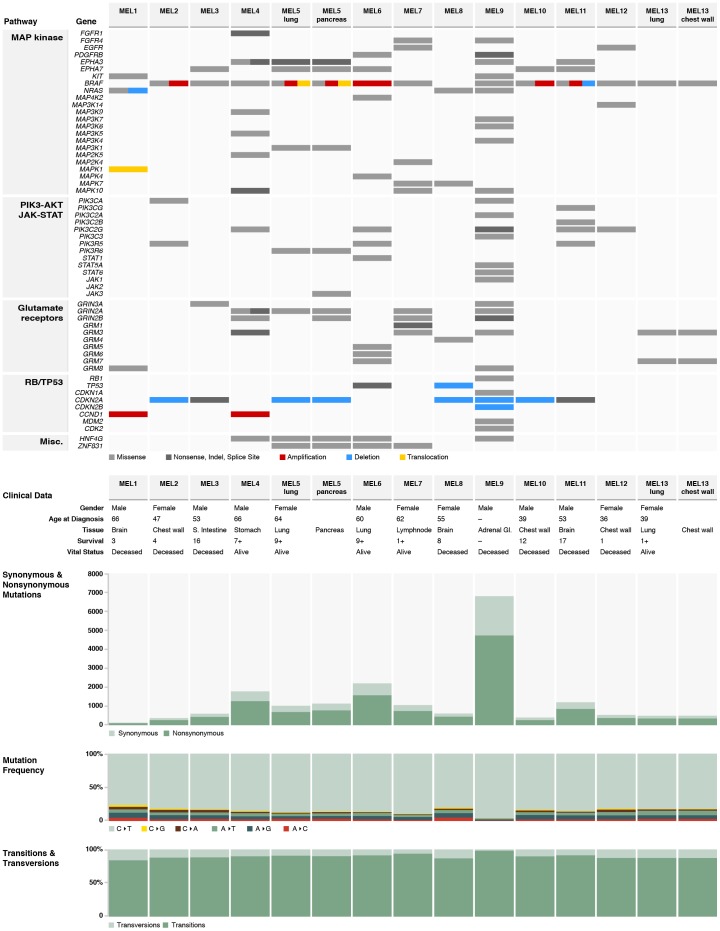
Mutation pattern, spectrum, and clinical features in 15 metastases from 13 WGS melanoma cases. Mutations found in genes from MAP kinase, PI3K-AKT, RB/TP53 pathways and glutamate receptors are shown. Copy number alterations and structural variants found in *BRAF, NRAS, TP53, CDKN2A/2B*, and *CCND1* are also displayed. The numbers and frequencies of tier 1 transition and transversion events identified in all 15 tumors are shown.

All 13 WGS patients, from whom 15 tumors and 13 sets of normal PBMC were included in this study, had stage IV melanoma. The metastatic samples were from diverse locations including lung, chest wall, brain, lymph node, stomach, small intestine, and adrenal gland (**Figure 1**). Using an Illumina paired-end sequencing strategy, tumor and normal genomes were sequenced to at least 29.5X-fold and 35X-fold haploid coverage, respectively, with corresponding diploid coverage of 98.84% or better based on concordance with SNP array data (**Table S1 in File S1**). Candidate somatic changes were predicted using multiple algorithms [21]–[25] and selected for hybridization capture-based validation (Supplementary Materials and Methods). We included capture probes corresponding to all putative somatic single nucleotide variants (SNVs) and small insertions/deletions (indels) that overlap with coding exons, splice sites, and RNA genes (tier 1), a number of high-confidence SNVs and indels in non-coding conserved or regulatory regions (tier 2), and non-repetitive regions of the human genome (tier 3). In addition, we included predicted somatic structural variants (SVs) genome-wide for validation (Methods1). Analysis of the high depth sequencing data resulting from the captured target DNAs of 15 tumor and 13 normal samples (**Table S2, S3** in File S1 and Supplementary Materials and Methods in File S1) confirmed 17,361 tier 1 point mutations, with a validation rate of 93.6%, 84 tier 1 indels, and 411 somatic SVs (**Tables S4 and S5 in File S2**). Seven of 15 tumors had over 1,000 tier 1 SNVs. This is among the highest mutation frequency of known cancers. For comparison, AMLs have a median of 13 tier 1 changes per genome [cite TCGA AML] [26] and metastatic breast cancers have been reported with between 32 (lobular) [27] and 50 (basal-like) [28]. C->T transitions were predominant in all 15 tumors, consistent with a UV damage signature (**Figure 1**). Notably, MEL9, with 6,795 validated tier 1 point mutations (7.7-fold times the average number from the other 12 cases), exhibited the highest C->T transition rate among 15 tumors. The patterns of point mutations were very similar for the paired metachronous tumors from patients 5 and 13 (**Figure 1** **and Table S6a in File S2**). Notably, we identified 443 tier 1 dinucleotide mutations in 13 WGS cases and among them, an average of 74% (ranging from 68% to 78%) are CC->TT changes, consistent with previous reports [11]. The ratio between dinucleotide and point mutations in Tier 1 ranges from 0.76% to 6.28% while the ratio in Tiers 1–3 ranges from 0.46% to 2.43%, consistent with the higher GC content in the coding sequences. (**Table S7 in File S1**)

We validated 84 coding small indels (65 deletions and 19 insertions) ranging from 1 to 37 base pairs (bp) in length, including a complex frameshift indel (4 bp deletion and 2 bp insertion) in *TP53* and 1 bp deletions in *STAG2* and *CDKN2A* (**Table S5 in File S2**). We also identified a total of 411 validated rearrangements for the 13 cases (range 10–87), including 240 deletions, 95 inversions, and 69 translocations (**Table S5 in File S2**). Across all 15 tumors, there was a median of 11 chromosomal rearrangements disrupting protein-coding regions per tumor. Recurrent deletions in tumor suppressor *FHIT*, the fragile histidine triad gene, were identified in MEL5 and MEL10, while large deletions interrupting *CDKN2A* were identified in 5 tumors from 4 patients (MEL2, 5, 8, 9, and 10). Further, one 1 bp deletion and one nonsense mutation in *CDKN2A* were identified in MEL3 and MEL11, respectively. A deletion involving *CTNNA2* was also detected in MEL10. A focal amplification of *CCND1* was observed in MEL1 and MEL4, which resulted in increased expression levels, according to DNA microarray analysis from those tumors (data not shown). SVs and copy number alterations for each of the 15 sequenced tumors are shown in **Figure S1** in File S1, providing a comprehensive view of genetic aberrations in melanoma metastases.

Previous studies showed that *TERT* promoter mutations are frequent in familial and sporadic melanoma [29], [30] and other cancer types [31]. We identified somatic mutations within the promoter of *TERT* in 9 of 13 (68%) melanoma cases. Four distinct somatic base substitutions were observed G>A, 101–146 bases upstream of the *TERT* transcription start site (TSS). Three are point mutations (C205T (MEL8), C228T (MEL4, 5, 10, 11), and C250T (MEL1, 8, 9, 12)) and one is a dinucleotide mutation (C242T, C243T (MEL13)) (**Table S6b in File S2**). Sequence coverage levels achieved around the *TERT* promoter region (500bp upstream from TSS) were low (∼7.4X on average) due to the sequence context (high GC) and repeat content. It is therefore possible that the prevalence of *TERT* promoter mutation could be higher than 68% in our sample set.

### Significantly mutated genes (SMG) and pathways in melanoma

**Figure 2:**
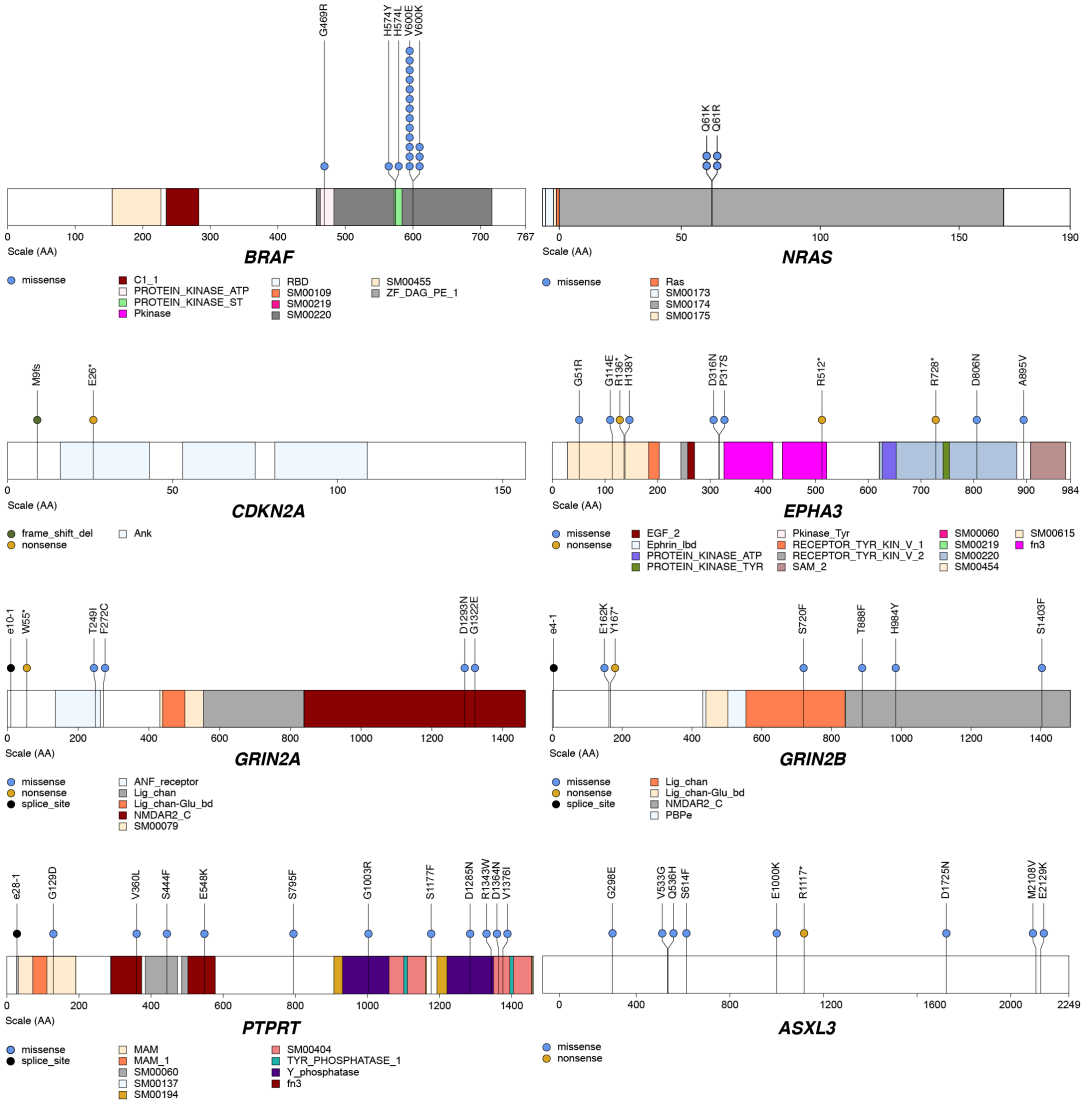
Mutation distribution in *BRAF*, *NRAS*, *CDKN2A*, *EPHA3*, *GRIN2A*, *GRIN2B*, *PTPRT*, and *ASXL3*. The locations of conserved protein domains are highlighted. Each nonsynonymous substitution, splice site mutation, or indel is designated with a circle at the representative protein position with color to indicate the translational effects of the mutation.

After our initial discovery using 13 WGS cases followed by the validation analysis described above, we performed further extension screening in 15 melanoma cases (25 metastatic tumors and matched normal tissue; 6 cases with multiple metastases). 1,209 genes were chosen for screening based on our initial WGS results and mutations and genes reported in several recent genomic studies of melanoma (**Tables S8a and S8b in File S2**) [13], [15]–[18]. Using mutations identified in all 28 cases, we performed MuSiC [32] analysis to discover genes displaying significantly higher mutation rates than expected based on the background mutation rate. A small group of genes was identified as significant after applying a 5% false discovery rate threshold (**Table 1**). This group included *BRAF* and *NRAS*, which were found to be mutated in 18 and 4 patients of 28, respectively (**Figure 2**). MEL9, the adrenal gland metastasis that was hypermutated, harbored mutations in both *BRAF* (H574L) and *NRAS* (Q61R); these two mutations were found to be present in the same variant allele frequency cluster (see Subclonal architecture in melanoma below). Meanwhile, mutations were not detected in either *BRAF* or *NRAS* in MEL6, a lung metastasis, and also four other tumors from the latter discovery group of 15 cases. The SMG list includes other genes known to be potentially involved in cancer. For instance, protein tyrosine kinase *EPHA3*, known mostly for its role in lung cancer [33], had 7 missense and 3 nonsense mutations, and tumor suppressor *CDKN2A* harbored one splice site, one nonsense, and one frame-shift indel, respectively (**Figure 2**). Mutations in a wide variety of protein families were seen in this study, including a large number of non-synonymous mutations in protein tyrosine phosphatases (e.g., *PTPRB*, *PTPRT*, and *PTPN13*), and protein tyrosine kinases (e.g., *EPHA7*, *EPHA3*, *KIT*, *FGFR4*, *FGFR1*, and *ROS1*). Of note, 8 missense and 1 nonsense mutations were found in *ASXL3*, a member of the polycomb group. The existence of mutations in glutamate receptors was described in prior exome sequencing studies [16], and our data not only confirmed that *GRIN2A* was mutated in melanoma (5 out of 28 cases) but also showed that *GRIN2B* was recurrently mutated (**Figure 2**). In addition, a number of mutations have also been found in other metabotropic glutamate receptors, such as *GRM1* and *GRM3-8*. Specifically, out of 23 nonsynonymous mutations from GRM genes, one nonsense and four missense mutations were from *GRM3*, previously shown to harbor activating mutations in melanomas [17]. The observed mutation rate was 0.22 to 143 mutations per Mbp in the TCGA dataset compared to 3 to 155 mutations per Mbp in our 15 whole genome sequenced samples. In addition to the similar distribution of mutation rates, we also observed recurrent single nucleotide variants including S225F and G394E in EPHA7 and G114E and R136* in EPHA3 from both datasets. The Comparison of the number of mutations in significant genes between this study and TCGA report [34] is shown in Table S15 in File S1.

**Table 1:** Significantly mutated genes identified in 28 paired melanoma cases.

Gene	Indels	SNVs	Total Mutations	Mutated Cases	Mutation Frequency	Covered bps	Mutation per Mbp	P-value	FDR
***BRAF***	0	22	22	18	0.6429	62953	349.47	0	0
***NRAS***	0	4	4	4	0.1429	16408	243.78	2.1749E−07	0.00059482
***CDKN2A***	1	3	4	4	0.1429	16864	237.19	4.4204E−06	0.00743995
***EPHA3***	0	10	10	7	0.25	89708	111.47	2.1443E−05	0.02606568

Melanomas harbor a number of aberrantly regulated signaling pathways, including INK4A-CDK4/6-RB, ARF-TP53-MDM2, RAS-RAF-MAPK, PTEN-PI3K-AKT, and aMSH-MC1R-cAMP-MITF, all of which may be altered via genetic, genomic, or epigenetic mechanisms [35], [36]. Mutations and rearrangements were identified in *BRAF*, *NRAS*, and several MAP kinases including *MAP3K1*, *MAP4K2*, and *MAP3K14* in the MAPK signaling pathways; 24/28 patients had at least one mutation in this pathway. In addition, 18 patients harbored somatic alterations in genes affecting the RB/TP53 pathway, including *CDKN2A*, *CCND1*, *MDM2*, and *CDK2* (**Figure 1** **and Tables S9a, S9b, and S9c in File S2**). *GRM3* and *GRIN2A/2B* alterations were also frequently observed in the tumors sequenced herein, suggesting the importance of glutamate mediated transduction in melanoma (**Figures 1** **and** **2**).

### Recurrence analysis using 96 additional melanoma cases

In addition to the 15 paired targeted samples, we also screened 1,209 genes using an extension set of 97 melanoma tumor samples from 96 patients. Since matched normals are not available for these 96 patients, we focused our analysis on known mutations and truncation mutations. We identified 1,716 recurrent nonsynonymous mutations found previously in our paired discovery samples, in the COSMIC (Catalog of Somatic Mutations in Cancer) database [37], or in recent melanoma studies (**Table S10 in File S2**) [13], [15]–[18], as well as 1,287 truncation (nonsense, splice site, and frame-shift) variants in 616 genes (**Table S11 in File S2**). *BRAF* and *NRAS* mutations were identified in 60 and 19 patients, respectively. Additionally, three patients harbored mutations in both *BRAF* and *NRAS*; 10 patients exhibited dinucleotide polymorphisms (DNPs) in *BRAF*, and one DNP was identified in *NRAS*. Further analysis revealed a number of truncation mutations in well-established tumor suppressors (*TP53* and *RB1*), protein phosphatase genes (e.g., *PTEN*, *PTPRB*, *PTPRD*, *PTPRN2*, *PTPRT, and PPP1R3A*), and chromatin remodeling genes (e.g., *ASXL3*, *MLL2*, and *ARID2*) (**Figure S2** in File S1). In addition, three truncation variants (2 nonsense and 1 splice site) were found in the *PREX2* gene, consistent with previous findings (**Figure S2** in File S1) [19]. Our analysis using MuSiC [32] identified *ASXL3* and *PTPRT* as harboring co-occurring truncation mutations in our 96-patient extension set (P = 0.002). All three patients containing a *PREX2* truncation also had an *NRAS* mutation (P = 0.008 for the co-occurence); none of the three had a *BRAF* mutation (P = 0.062 for the mutual exclusion between *PREX2* and *BRAF*). *ASXL3* truncations also co-occurred with *NRAS* (P = 0.044). Interestingly, tumor suppressors *PTEN* and *RB1* co-occurred in our extension dataset (P = 0.019), harboring six and four truncation events, respectively.

### Subclonal architectures and driver mutations in melanoma

**Figure 3:**
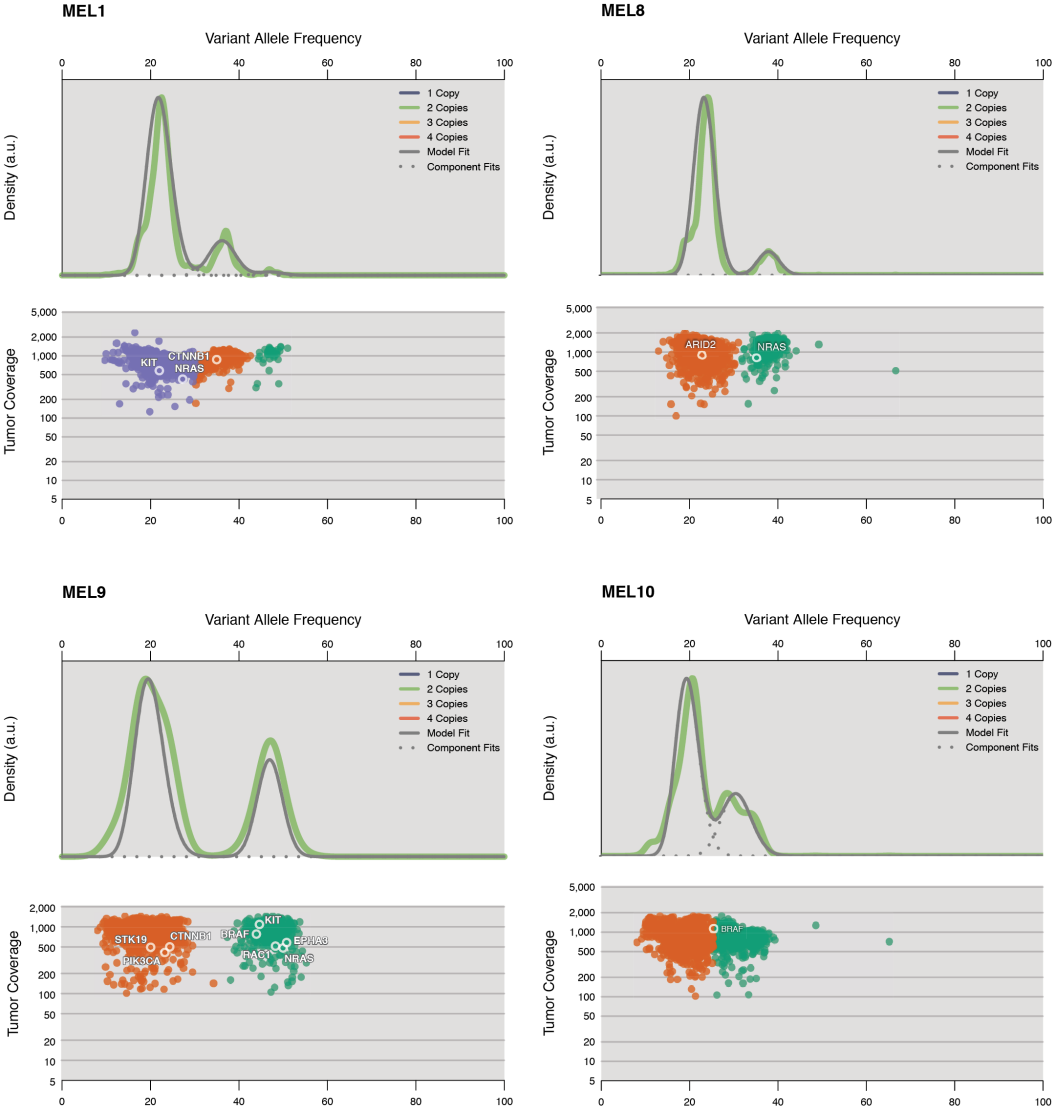
Overview of subclonal landscape in melanoma (MEL1, 8, 9, and 10) and their associated driver mutations. Two plots are shown for each case: kernel density (top), followed by the plot of tumor variant allele frequency by sequence depth for sites from selected copy number neutral regions (see Methods). Data shown are from chromosomes 1, 3, 4, 6, 10, and 13 for MEL1, from chromosomes 1, 2, 5, 12, and 20 for MEL8, from chromosomes 1, 3, 4, 6, 7, 12, and 13 for MEL9, and from chromosomes 6, 7, 10, 13, and 15 for MEL10. The data show evidence of two clusters in MEL1, MEL8, MEL9 and MEL10 with the majority of mutations from the lower allele frequency clusters. Mutations detected in significantly mutated genes in this study and genes implicated in Hodis *et al*. [18] were labeled.

We took hundreds of validated somatic mutations with read depths of hundreds to thousands from capture validation and applied the SciClone algorithm (https://github.com/genome/sciclone) to cluster mutations with similar allelic fractions. These clusters are indicative of distinct subclonal populations of tumor cells. Multiple subclones were observed in the majority of 15 WGS tumors (**Table 2**). Due to the high mutation rate and complex copy number landscape in melanoma, the boundaries of some clusters could not be clearly separated using genome-wide data. We then selected “stable” genomic regions based on LOH and CNV analyses using VarScan 2 [38] and used somatic mutations from these regions for plotting (**Figure 3** **and Table S12a in File S2**). In MEL1, two distinct clusters at 36.7% and 21.7% Variant Allele Frequency (VAF)s were identified. The majority of mutations were from the 21.7% VAF cluster. MEL8 displayed a similar pattern as MEL1, with one cluster at 37.8% VAF and another cluster at 23.4%. The hypermutated MEL9 tumor has the founding cluster at 46.8% VAF and the secondary but dominant cluster centered at 19.8%, suggesting that a massive mutation expansion took place in the 17.6% VAF cluster. Likewise, MEL10 had two clusters, centered at 30.6% and 19.5% VAFs respectively (**Figure 3**). These estimates of tumor heterogeneity represent a lower bound, and it is possible that additional subclone(s) were present in these samples but not detected. Our results demonstrate that melanoma is a disease characterized by significant intra-tumor heterogeneity.

**Table 2:** Clonal numbers and variant allele frequencies for driver mutations in 15 WGS metastatic tumors.

Sample	BRAF VAF	NRAS VAF	Focal Amplification of BRAF and/or NRAS	CN for BRAF	Number of subclones	Founding cluster frequency	Subclone cluster frequency
**MEL1**	NA	(Q61R) 26.89%	No	2	2	36.69	21.74
**MEL2**	56.88%	NA	Yes	3.78	2	46.26	25.31
**MEL3**	32.31%	NA	No	2	2	29.02	19.9
**MEL4**	28.35%	NA	No	2	2	23.53	12.55
**MEL5 (lung metastasis)**	(V600K)67%(WGS -122 reads)	NA	Yes	5.26	2	22.24	14.32
**MEL5 (pancreas metastasis)**	(V600K)99%(WGS- 181 reads)	NA	Yes	9.67	2	39.41	45.6
**MEL6**	NA	NA	Yes	2.59	1	26.33	NA
**MEL7**	32.34%	NA	No	2	1	28.08	NA
**MEL8**	NA	(Q61K)34.58%	No	1.3	2	37.77	23.37
**MEL9**	43.30%	58.17%	No	2	2	46.8	19.81
**MEL10**	(V600E) 25.31%	NA	Yes	2.97	2	30.62	19.47
**MEL11**	70%(WGS-54 reads)	NA	Yes	2.64	2	41.07	23.95
**MEL12**	58.44%	NA	No	2	2	32.85	19.27
**MEL13 (lung metastasis)**	25.36%	NA	No	2	1	30.2	NA
**MEL13 (chestwall metastasis)**	32.25%	NA	No	2	1	32.38	NA

By associating mutations with specific subclonal populations, we can infer the relative order in which these mutations were acquired. 12 of 13 *NRAS* and *BRAF* mutations in the cohort are clonal (or lie in copy number amplified regions), suggesting that these mutations are involved in melanoma initiation (**Figure 3** **and** **Table 2**). In MEL1, our analysis showed that *CTNNB1* T41I missense (a highly recurrent site) maps to the founding clone while *KIT* P157S (a novel mutation) resides in a subclone, suggesting the former was an early event, while the latter may have contributed to progression. Similarly, *NRAS* Q61K is present in all cells of MEL8, suggesting an initiation role, while the *ARID2* S1382F missense mutation is subclonal (**Figure 3**). MEL9 is characterized by both *NRAS* and *BRAF* mutations in founding clone, along with a *CTNNB1* P16S mutation that arose later in the evolution of the tumor.

### Distinct mutational signatures in founding and secondary clones of a hypermutated sample (MEL9)

**Figure 4:**
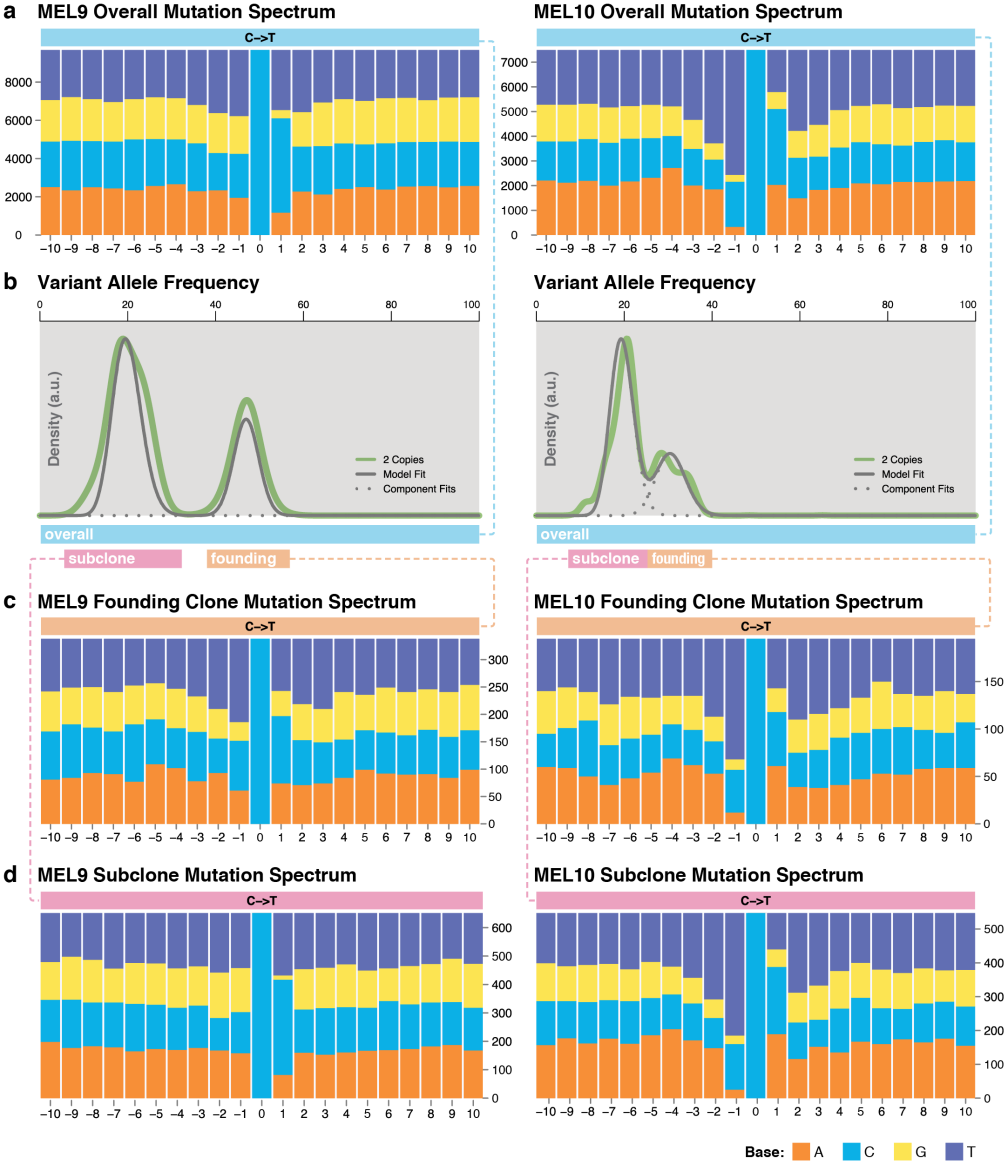
Dissecting mutational mechanisms using subclonal mutations. (a) Overall sequence context surrounding C->T transitions in MEL9 and MEL10. (b) Density plots showing the founding clone and subclone in MEL9 and MEL10. (c) Mutation context analysis of the founding clones detected a UV mutation signature in both MEL9 and MEL10. (d) Mutation context analysis of secondary clones detected a UV signature in MEL10 but not MEL9.

It has been shown that DNA damage caused by UV light often leads to the formation of covalent links between two adjacent pyrimidine residues [11]. As a result, C->T mutations in melanoma samples often occur at dipyrimidine sequences. Our analysis of 13 WGS melanoma cases showed that 12 cases had greater than 89.9% of C->T mutations occurring at the 3′ base of a pyrimidine dinucleotide, supporting previous findings [11]. However, MEL9, the hypermutated tumor, lacks this signature and has only 59.5% C->T occurring at the 3′ base of a dipyrimidine, comparable to 53% expected by chance (**Figure 4**). We reasoned that the UV signature in MEL9 might be masked by a large number of subsequent mutations arising from some other mechanism. One candidate was that these mutations were the result of a DNA repair defect (e.g., S418F and G1134R in *MSH6*, G2569S in *BRCA2*, or G648E in *ERCC6*). To test this hypothesis, we independently analyzed mutations from the founding and subclonal populations described above. (**Figure 4**). Strikingly, these two subclones in MEL9 exhibited two very distinct phenotypes. The founding clone exhibited a classic UV-damage phenotype with an abundance of C->T transitions and a disproportionately higher number of pyrimidine bases preceding the mutated cytosine bases (Proportion test P = 1.60×10^−10^). (**Figure 4**). In contrast, the subclone exhibited a typical pyrimidine base frequency preceding the mutated C base (59.5%, P = 0.17); interestingly this subclone had a significantly higher frequency of pyrimidine bases following the mutated C base (P = 1.72×10^−40^), consistent with findings in another hypermutated melanoma reported by Berger *et al.* [*19*]. Our hypothesis is that UV-driven mutations in the originating, founding clone of MEL9 damaged a DNA-repair gene and spurred a massive deficit in DNA repair. The resulting large number of mutations, occurring later than the UV damage, make up the lower-VAF subclone. The mutation context observed in the secondary clone of MEL9 does not match the patterns expected from defects in *MSH2* and MSH6, and it may be attributable to another repair pathway. As a control, we also dissected the mutation spectrum in the founding clone and subclone of MEL10. The subclone for MEL10 also has a larger number of mutations, (**Figure 4**) but both show a typical UV-signature, with a significant number of Cytosine and Thymine bases preceding C->T transition sites (P-value for founding clone  = 4.01×10^−21^, P-value for secondary clone  = 3.83×10^−65^).

### Clonal and phylogenetic relationship among metastatic tumors from different sites

**Figure 5:**
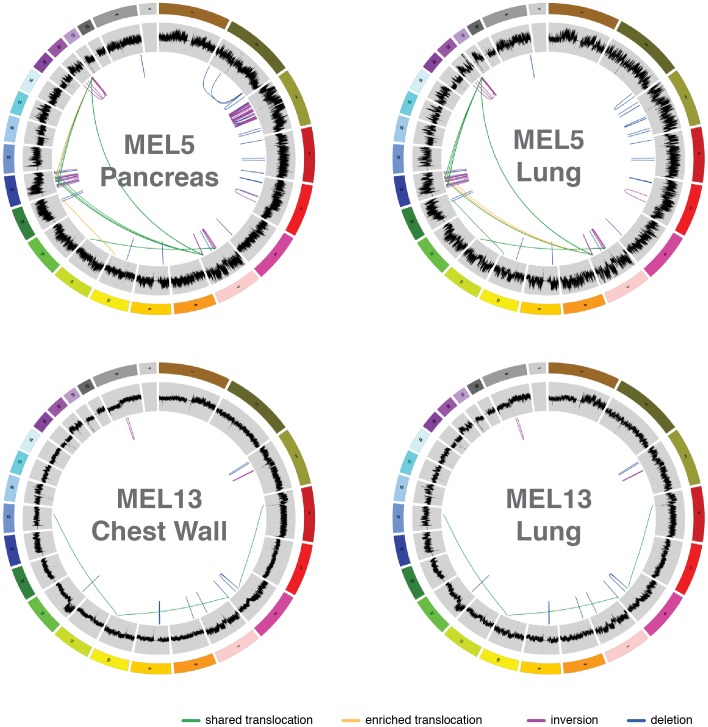
Comparison of Circos plots of the metastatic samples from two tissue sites of the same individuals (MEL5 and MEL13). In MEL5, pancreas tumor specific structural variants (inversions and deletions) are clustered on chromosomes 2 and 5, and pancreas or lung enriched rearrangements are drawn in yellow. In MEL13, highly similar copy number and structural variant patterns between lung and chest wall metastases are shown. No purity-based copy number corrections were used for plotting copy number.

**Figure 6:**
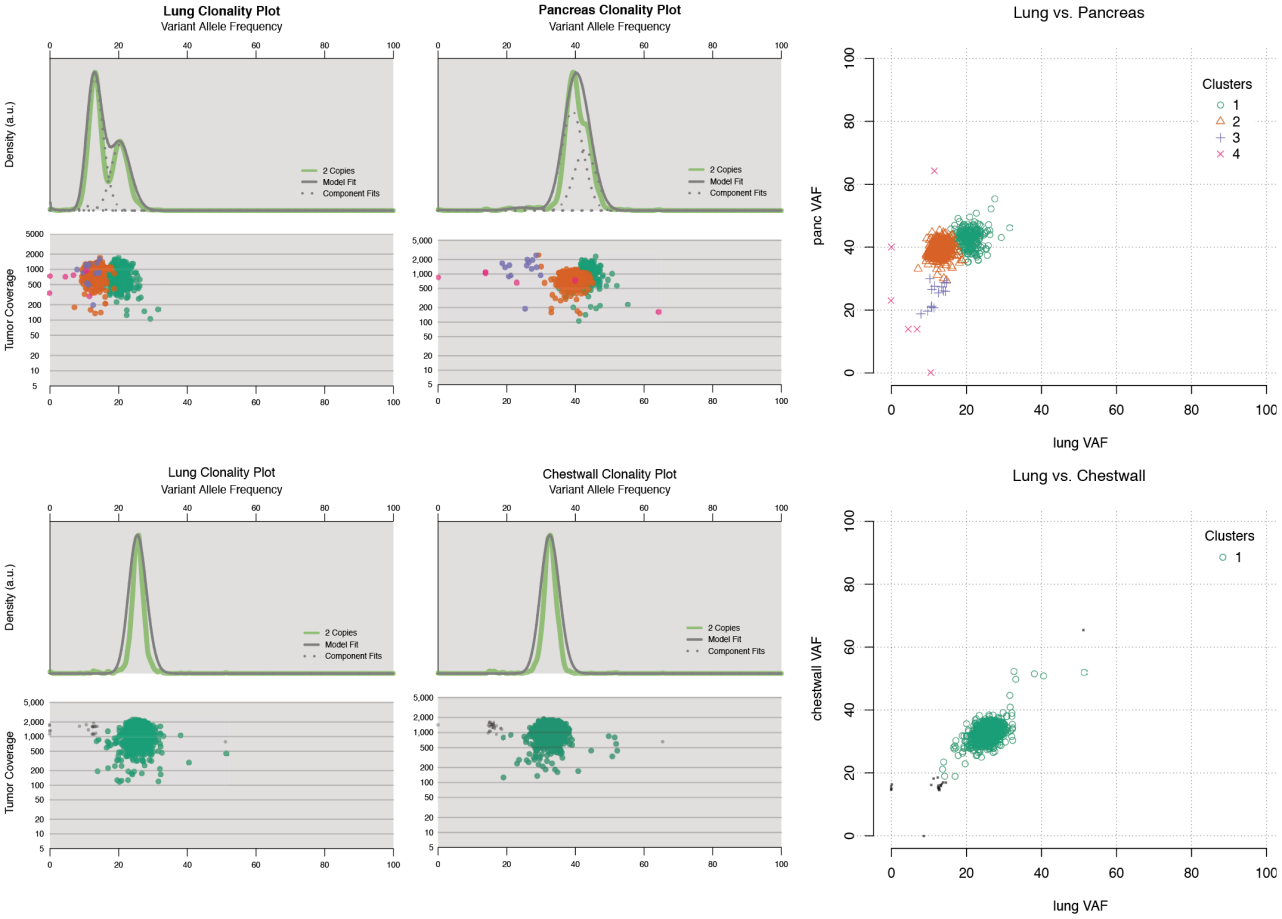
Comparison of clonality patterns of metastatic samples from two tissue sites of the same individuals (MEL5 and MEL13). Kernel density and variant allele frequency by sequence depth plots for each metastasis in MEL5 and MEL13. Data shown are from chromosomes 17, 18, and 21 for MEL5, and from chromosomes 3, 7, and 14 for MEL13. The plots indicate multiple clones in MEL5 with enrichment from lung to pancreas in MEL5, and nearly identical clonal pattern in both metastases in MEL13.

Among the 28 paired cases, 8 (2 WGS and 6 extension cases) had multiple metastasis samples, allowing the examination of relationships of different tumors from the same individual. First, we investigated the two WGS cases (MEL5 and MEL13) with two metastasis samples each. The rearrangement and copy number patterns were almost identical for the MEL13 paired metachronous tumors from chest wall and lung (**Figure 5**). In MEL5, a significant number of inversions on chromosome 3 were found to be present in the pancreatic metastasis but not the lung metastasis (**Figure 5**). Clonality analysis using point mutations from selected copy number neutral regions revealed at least two clusters each in the lung (22.2% and 14.3%) and pancreas (45.6% and 39.4%) metastasis from MEL5 (**Figure 6** **and Table S12b–c in File S2**). The MEL5 lung metastasis has two distinct mutation clusters, while the pancreas metastasis harbors two clusters with overlapping boundaries. A comparison of genome-wide tier 1 mutations in the pancreas versus lung metastases (**Figure 6** **and Table S12f in File S2**) shows that greater than 99% of the tier 1 mutations (1127/1139) are shared between these two samples, indicating they likely emerged from the same progenitor clone in the primary tumor. Many MEL5 mutations appear to be enriched in the pancreas sample. Additionally, a wider range of VAFs present in the pancreas sample indicates that numerous copy number altering events occurred after the initial development of the pancreas metastasis. Both the lung and chest wall metastatic tumors in MEL13 have a very similar clonal pattern (**Figure 6** **and Table S12d–e in File S2**), with one dominant higher VAF peak (24.9% and 32.4%, respectively) shouldered by less distinct clusters of mutations. The slight difference in the peak VAFs of the dominant clusters in these two metastases suggests that the chest wall biological specimen has a higher purity (64.8% tumor) than that of the lung metastasis (49.8% tumor). Consistent with the kernel density plots (**Figure 6**), an analysis of VAFs of genome-wide tier 1 mutations in MEL13 (**Figure 6** **and Table S12g in File S2**) shows similar clonal architecture in the two metastasis samples, as most occur at comparable VAFs. Again, over 99% of the mutations are shared between the two metastases (503 of 505) in MEL13, with only 2 mutations being sample specific. This result suggests that both MEL13 metastases are derived from the same clone/subclone in the primary tumor.

**Figure 7:**
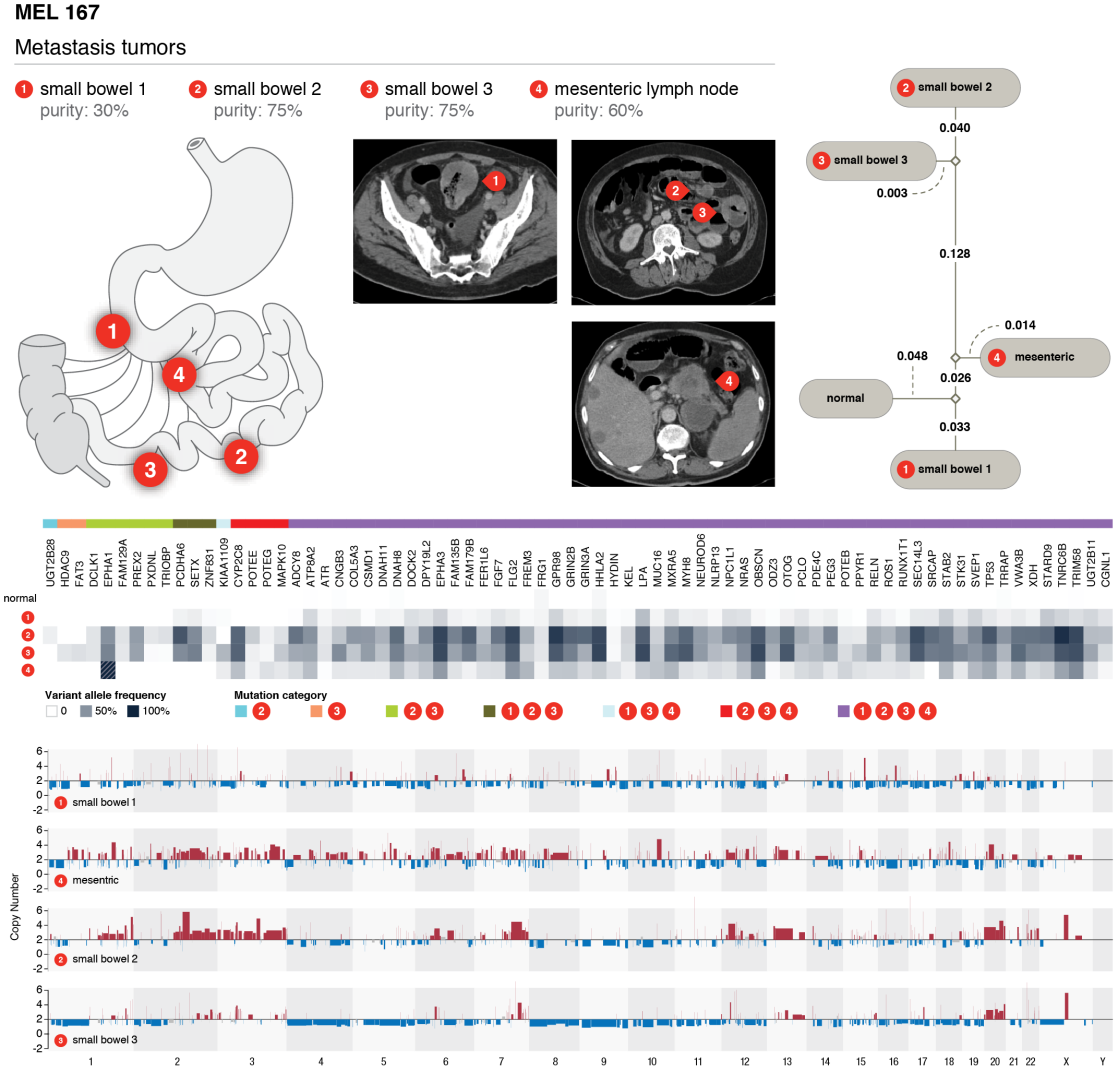
Phylogenetic and mutational relationships among four metastatic samples from different sites of the same individuals (MEL 167). Geographic locations and CT scans of metastasis samples in MEL167 with three tumor samples from small bowel (mass 1, mass 2, and mass 3) and one from mensenteric lymph node. Phylogenetic relationships, mutation patterns, and copy number landscape in all four tumors were shown. Purity based VAF corrections were applied prior to phylogenetic analysis.

Two cases (MEL167 and MEL174) with 4 synchronous metastatic samples were sequenced with a targeted extension panel. MEL167 has three tumor samples from small bowel and one from a lymph node. The relative locations of small bowel tumors 1, 2, and 3 from MEL167 are shown in **Figure 7**. Copy number analysis using VarScan 2 shows that small bowel tumors 2 and 3 share amplifications on chromosomes 7q, 13q, and 20, consistent with their close proximity in location. The lymph node tumor shared amplifications on chromosomes 2 and 3 with small bowel tumor 2 while small bowel tumor 1 showed no major amplifications genome-wide (**Figure 7**). Phylip-based phylogenetics analysis (Supplementary Materials and Methods in File S1) using mutations and their purity-corrected frequencies (**Table S13 In File S1 and Figure S3** in File S1) recapitulated the copy number analysis-based findings and identified small bowel tumors 2 and 3 as closely related and most divergent from the normal. The lymph node and small bowel tumor 1 are more similar to each other and appear to be less divergent from the the normal tissue (**Figure 7**). Mutations in *TP53* (H179Y), *NRAS* (Q61K), *ATR* (G2120C), and *EPHA3* (P317S) are shared by all four tumors, suggesting they are likely founding mutations from the primary tumor (**Figure 7** **and Table S14 in File S2**). On the other hand, *PREX2* (P614S) and *ZNF831* (P1639S) are only present in some tumors.

**Figure 8:**
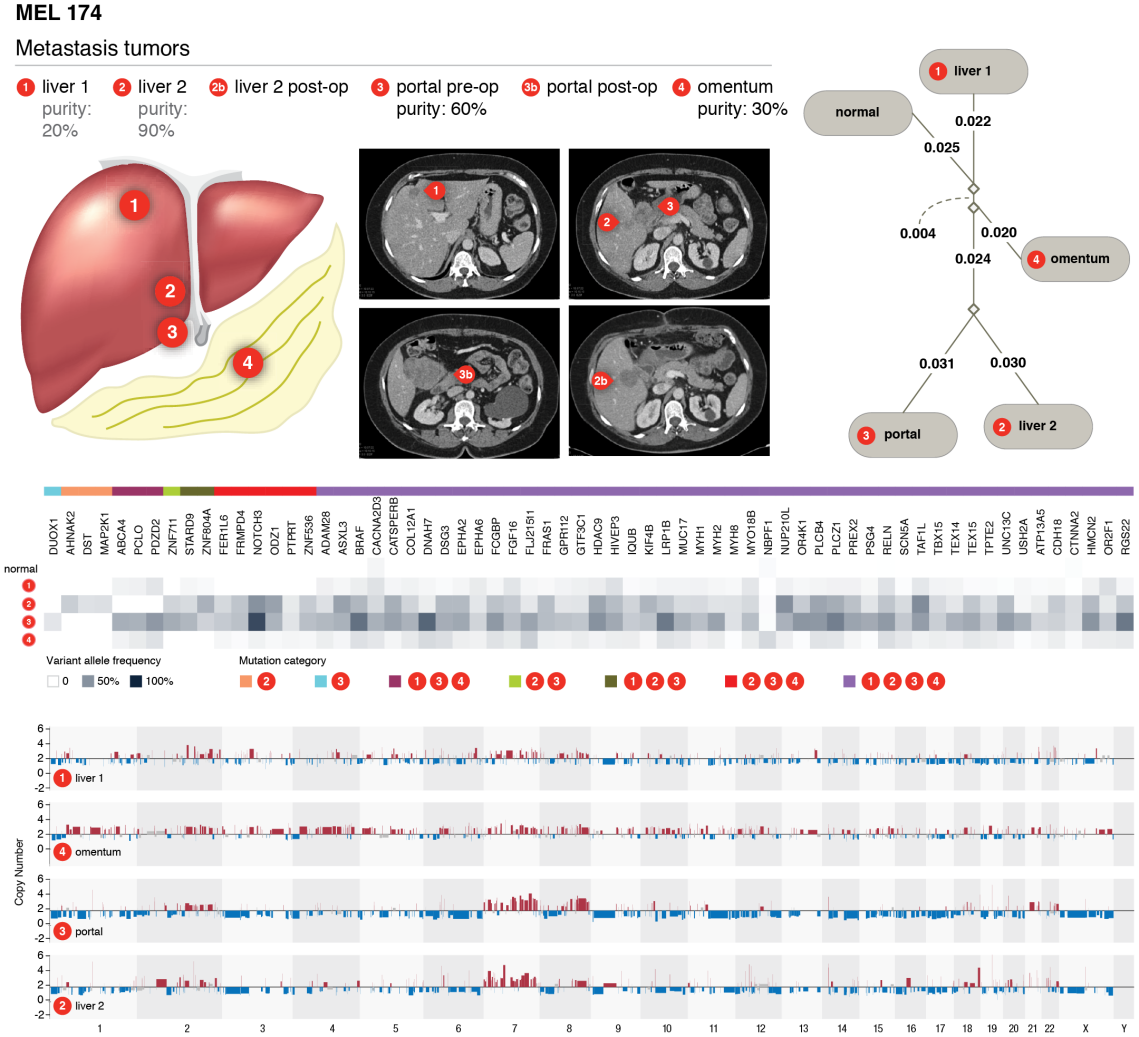
Phylogenetic and mutational relationships among four metastatic samples from different sites of the same individuals (MEL 174). Geographic locations and CT scans of metastasis samples in MEL 174 with 2 samples from liver (liver tumor 1, liver tumor 2), one from omentum, and one from portal nodule. Phylogenetic relationships among 4 metastatic tumors were shown. Purity based VAF corrections were applied prior to phylogenetic analysis. Increased variant allele frequency of BRAF (V600E) in liver 2 and portal module, both tumors showed resistance to vemurafinib treatment. BRAF amplifications were also shown in liver 2 and portal module tumors.

We also analyzed a quartet of synchronous tumors from MEL174: two from the liver (liver tumors 1 and 2), one omentum tumor, and one portal nodule tumor (**Figure 8**). Liver tumor 2 shows some similarities to the portal nodule tumor, with both having major amplifications on chromosomes 7 and 8, while liver tumor 1 and omentum tumor show a “quieter” overall copy number landscape. Copy number alterations and mutation-based phylogenetic largely agree on the relationship between these tumors (**Table S13** in File S1 **and Figure S3 in File S1**). Liver tumor 1 and the omentum tumor responded to treatment with the BRAF inhibitor vemurafinib, while the others continued growing/progressing during treatment. We found that both *BRAF* (V600E) and *CTNNA2* (R755*) have much higher variant/mutant allele frequencies in the non-reponsive portal nodule (*BRAF*: 65.2%; *CTNNA2*: 15.6%) and liver tumor 2 (33.3%; 16.1%) than in the omentum nodule (8.2%; 1.1%) and liver tumor 1(14.3%; 2.8%). The *CTNNA2* mutation is almost undetectable in the omentum nodule with only one read, out of 94 total reads, supporting the mutant allele. A *MAP2K1* (E203K) mutation is only detected in liver tumor 2 despite the high coverage (>40X) in all 4 metastases (**Table S14 in File S2**). After applying purity-based VAF adjustments, the portal nodule has the highest adjusted VAF at 72.47%, followed by liver tumor 2, liver tumor 1, omentum nodule at 55.55%, 47.63%, and 41% respectively. Our data indicate that sample-specific genetic alterations and variable frequencies of mutations might contribute to differential treatment responses among metastasis samples from the same patients, consistent with a previous report [39] (**Figure 8**). Moreover, our analysis shows that all four metastatic tumors from both patients were derived from the same primary tumor but their patterns of mutations diverged considerably during evolution and metastatic growth.

## Discussion

This study represents a comprehensive whole genome and targeted sequencing analysis of 124 human melanoma cases, including 13 WGS, 15 paired targeted, and 96 unpaired targeted extension cases. Besides the expected coding mutations in *BRAF*, *NRAS*, *CDKN2A*, and other genes detected, significant numbers of recurrent copy number variants and structural rearrangements found in this analysis of 13 WGS cases suggest that they may be important initiating and metastatic events in melanoma. Examples include the amplification of *CCND1* on chromosome 11 in two patients, and *CDKN2A* deletions, which were observed in 5 of 13 patients, confirming previous data [40], [41]. Our *in silico* significance and proximity analysis of 28 paired cases (13 WGS and 15 targeted cases) identified known (e.g., *BRAF*, *NRAS*, *CDKN2A*, and *GRIN2A*) and novel (e.g., *EPHA3, GRIN2B, and ASXL3*) genes involved in melanoma (**Table 1** **and** **Figure 2**). Moreover, our extension analysis of 96 patients using targeted sequencing of 1,209 genes, revealed a number of truncation mutations in *TP53*, *RB1*, *PTEN*, *PTPRD*, *ARID2*, *ASXL3*, and other genes co-occuring with *BRAF* and *NRAS* mutations, suggesting their cooperating roles during the progression of melanoma. These alterations in other pathways, such as the PI3K pathway, may partially explain the resistance of *BRAF*-mutant melanoma to RAF inhibitors.

Previous melanoma whole genome [11], [19], [42] and whole exome [18], [43], [44] studies have uncovered a complex landscape of melanoma genomes including high mutation rate, a complex copy number landscape, predominance of UV-related C>T transitions, and frequent genetic alterations in well-known drivers of melanomagenesis such as *BRAF, NRAS, TP53, CDKN2A*, and *PTEN*, which was corroborated in our study.

As described, all 13 whole genome analyzed cases harbored mutations or copy number alterations in either *NRAS* or *BRAF* (with 1 tumor having both). Their presence largely in the founding clone, as determined by VAF, is evidence of the contribution of these genomic changes to melanoma initiation. Interestingly, WGS of these tumors identified the mutations, copy number alterations, and structural variants that occurred concomitantly with these previously identified “driver” mutations. These findings provide a mutational profile of melanoma metastasis against the background of an initiating oncogenic event and point to genomic changes that may be implicated in the loss of senescence and oncogenic transformation in melanoma. In addition, the predominance of C->T transitions provides impressive evidence of UV-related DNA damage in WGS metastatic tumors and suggests that many of the potential driver SNVs were present long before the spread of metastatic disease occurred. The sheer number of SNVs in these tumors is striking, with one tumor possessing over 6,000 tier 1 mutations (∼180 mutations per Mbp), and the average of greater than 1,300 tier 1 SNVs per tumor constitutes a large number by any metric.

In this study, we focused on subclone structures and intra-tumor heterogeneity of melanoma, which was not addressed in previous studies. Our results for 15 metastatic melanoma tumors demonstrate that melanoma is mainly a multi-clonal disease which harbors diverse numbers of clonal populations with various frequencies and densities. In contrast to point mutations, which are likely the result of UV damage, the rate of structural variation in this study was similar to that previously described for most malignancies. Whole genome sequencing data herein indicate that complex rearrangements may generate important gain- and loss-of-function driver events in melanoma oncogenesis. Moreover, many rearrangements may occur preferentially in genes that are spatially localized together within transcriptional or chromatin compartments, perhaps initiated by DNA strand breaks and erroneous repair.

Subclone-specific mutation signature analysis in a hypermutator sample (MEL9) revealed that the founding clone and the subclone displayed a distinct mutation context, suggesting different mutational mechanisms for two subclones from the same tumor. The vast majority of mutations and re-arrangements were shared between the two metastatic samples for both cases with metachronous tumors, suggesting they are derived from the same subclone(s) of the primary tumor. A significant number of pancreas-specific inversions were identified in MEL5, though their potential role in the progression of the disease is unclear.

Finally, in studying the clonal architecture of all WGS cases, we confirmed that clonal heterogeneity is a common phenomenon in melanoma and driver initiation and progression mutations are all required for the development of melanoma. This suggests that knowing the clonal architecture of each patient's tumor will be essential for understanding the evolutionary history of each tumor and for formulating optimized treatment options. Importantly, our phylogenetic study of two patients each with 4 different synchronous metastases clearly revealed the complex relationships among these tumors derived from the originating primary tumor and suggest that melanoma therapy is a moving target and constant monitoring of tumor genomes may be required to develop an effective treatment plan as evidenced by the differential response to Vemurafenib of 4 synchronous metastases from MEL174.

## Methods

### Illumina library construction and sequencing

The procedure described by Mardis *et al* [*45*] was followed for library construction and sequencing. Briefly, Illumina DNA sequencing was used to generate between 117 and 286 million base pairs of sequence data for each of the 15 metastatic tumors and 13 matched normal samples, with haploid coverages ranging from 29.51 to 63.22 (Table S1 in File S1). Comparison of heterozygous SNPs detected in the whole genome sequencing (WGS) data with SNPs array genotypes confirmed bi-allelic detection of between 98.7 and 99.7% of the heterozygous array SNPs in the 13 cases. Detailed coverage statistics for all cases are included in Table S1 in File S1.

### Mutation detection pipeline

For each sample, reads were aligned using BWA 0.5.9 (http://sourceforge.net/projects/bio-bwa/) on a per-lane basis, merged into a single bam file, and duplicate reads were removed using Picard 1.29 (http://picard.sourceforge.net). Sample variants were called using Samtools (svn rev 599) [^23^]. Somatic single nucleotide variants were detected using SomaticSniper [^25^]. High quality somatic predictions were defined as those sites with a SomaticSniper somatic score greater than 40 and an average mapping quality greater than 40. Indels in all samples were called using a combination of Pindel [^22^] and GATK [^21^]. Somatic variants were grouped into tiers based on genome annotation as described previously [^45^]. Y chromosome variants were filtered for all female patients.

### Structural variant detection

Structural variants (SVs) in all samples were predicted by BreakDancer [^24^] and SquareDancer (https://github.com/genome/gms-core/blob/master/lib/perl/Genome/Model/Tools/Sv/SquareDancer.pl). All SV predictions were filtered using TIGRA (Chen et al., in preparation) to identify assembled breakpoints in SV flanking reads. The same procedure as described in Ding *et al.* [^26^] for selecting somatic SVs was used.

6491 structural variants from the 13 WGS cases were sent for capture validation

### Validation of structural variants

All BWA-aligned capture reads and their mates that map within 1000 bp of the structural variant breakpoints were realigned by CrossMatch (version 1.080721) to the assembled SV contigs and to the reference. The threshold for an acceptable alignment is < = 1 unaligned base at either end, < = 1% substitutions, < = 1% indels and a CrossMatch score > = 50. An SV-supporting read is required to span the breakpoint on the SV contig, align to 10 bases of flanking on each side of the breakpoint, and have no alignment to the reference above minimum alignment criteria. SV-supporting reads were tabulated in the tumor and normal sample separately, and a Fisher's Exact test was applied to these counts to determine the somatic status of each variant. The same method for determining SV-supporting reads was applied to the WGS alignment data for those calls deemed somatic via all other criteria. Variants with any SV-supporting reads in the normal WGS sample were filtered out as potential germline variants or alignment artifacts. An additional filter was put in place to filter ALU sequences and the remaining high confidence SV events were manually reviewed based on BWA mapping of supporting capture validation data to the assembled SV contigs spanning the breakpoint. 411 structural variants from the 13 WGS cases passed the final manual review and filtering.

### Kernel density analysis for identifying clusters and estimating allele frequencies for each tumor

Tumor clonality estimates were determined using the mutation allele frequencies from sites with deep coverage from capture validation data. To minimize the effect of coverage on allele frequency estimations, only mutations with >100x coverage in both the normal and tumor validation data were included in this analysis. Efforts were made to exclude somatic SNVs from regions containing copy number alterations identified in WGS data. Varscan 2 was utilized on whole-genome sequencing data to eliminate all LOH SNV calls. For each chromosome, the variant allele frequencies were plotted from both the tumor and normal samples at sites where the normal sample's variant allele frequency fell between 40% and 60%. Chromosomes where variants frequently exhibited highly variable variant allele frequencies in the tumor sample were excluded from the clonality analysis. Thus only a few diploid chromosomes were chosen to represent each sample for this analysis. The remaining SNVs were further segregated according to their segmented copy number states as predicted by cnvHMM (states of copy number equal to 2, 3, or 4), and each copy number state was analyzed individually. For each copy number state in the tumor, a kernel density estimate (KDE) plot was drawn for tumor variant allele frequencies using the density function in R. A customized R function evaluated each KDE plot to determine the number of significant peaks in variants existing in the copy number neutral, or diploid regions. These clusters thus identified served as an estimation of the number and relative composition of clones and subclones present in each tumor. Only copy number neutral data is presented in Figure 6.

### Significantly mutated gene analysis

We used the SMG test in the MuSiC suite^32^, using the particular options available for accommodating the large numbers of somatic mutations discovered in some melanoma cases. The SMG test identifies genes that have significantly higher somatic and germline mutation rates than background. To account for hypermutated samples, the ability to sub-group cases based upon similarities in their overall mutation counts was utilized, and P-values were calculated for mutated genes in each sub-group independently. All P-values were combined using the same methods as described in MuSiC suite^32^. For the purposes of this analysis, MEL4, MEL6, and MEL9 was considered as a hypermutant, with all other samples being placed in a separate sub-group for the SMG analysis. For the analysis of significantly mutated genes, genes not typically expressed in melanoma tumor samples were filtered if they had an average RPKM≤0.5. For the RNA-seq based gene expression analysis, we used the Pancan12 per-sample log2-RSEM matrix from doi:10.7303/syn1734155.1. A gene qualified as expressed if it had at least 3 reads in at least 70% of samples. For every gene, the average per-sample RNA-seq by Expectation Maximization (RSEM) value was calculated across TCGA SKCM samples from the same tumor-type.

### Proximity analysis

Validated somatic mutations were identified that clustered within specific protein regions across multiple individuals. This was accomplished by querying the distance between amino acids for every pair of mutations on a given transcript of a mutated gene within the sample set, and then determined which mutations fell within “close” proximity, where “close” was defined to be within a limit of 10 amino acids. We used the MuSiC suite^32^ for the proximity analysis.

## Supporting Information

File S1 **Supplementary Materials and Methods, along with Supplementary Figures S1 to S4 and Supplementary Tables S1 to S3, S7, S13 and S15 and references cited in the Supplementary materials and method section.** **Figure S1 in File S1**: Copy number patterns and structural variants identified in all 15 tumors sequenced. **Figure S2 in File S1**: Extension heatmap of recurrent *BRAF* and *NRAS* mutations as well as truncation mutations in selected tumors suppressor, protein phosphatase and chromatin remodeling genes. The mutual exclusion and co-occurrence of common recurrent drivers and truncation mutations are shown in the 96 extension cases. Three melanoma cases harbored both *BRAF* and *NRAS* mutations. **Figure S3 in File S1**: Density plots of variants to estimate the purity of the four metastases for each patient MEL167 and MEL174. Both the density plots and the Scatter plots were used for purity estimation of the metastases samples. **Figure S4 in File S1**: Flowchart showing the detailed overview of the analysis steps and the pipeline used for the analysis. **Table S1 in File S1**: WGS haploid coverage and SNP array concordance. Haploid and diploid coverage estimates are given for 15 whole-genome sequenced samples. Haploid coverage is calculated as the amount of non-redundant mapped read bases divided by the haploid size of the human genome. Diploid coverage is estimated from the fraction of heterozygous SNPs from high-density SNP array data that were present in SAMtools raw (unfiltered) or filtered SNP calls. **Table S2 in File S1**: Capture validation coverage. Custom capture validation coverage of putative somatic mutations is reported for the 13 cases in which such data were generated. Shown are the fraction of bases targeted that were covered >1x,>10x, and >20x in each sample. **Table S3 in File S1**: Tier 1–3 somatic SNVs predicted and validation rate. Numbers of validated somatic SNVs in tiers 1, 2, and 3 are shown for the 13 cases having both whole genome sequence data and custom capture validation. **Table S7 in File S1**: Dinucleotide polymorphisms (DNP) in 13 WGS cases. **Table S13 in File S1**: Purity estimation of the multi-metastases samples MEL167 and MEL174 using the density plots. **Table S15 in FileS1**: Comparison of the number of Tier1 SNVs in the TCGA melanoma dataset to the number of SNVs in the WGS dataset.
(DOCX)

File S2 **The Zipped file contains the Supplementary Tables S4, S5, S6, S8, S9, S10, S11, S12, S14.** **Table S4 in File S2**: Validated somatic point mutations and indels: See separate.xlsx file. **Table S5 in File S2**: Validated somatic structural variants: See separate.xlsx file. Validated structural variants in 15 whole genome sequenced samples are listed with Patient ID, chromosomal positions of each breakpoint (A, B), the type of event (DEL deletion, CTX translocation, INV inversion, INS insertion, ITX tandem duplication), and the size of event in bp. If a breakpoint is within a gene, the gene and transcript name are given with the direction of transcription and transcript substructure where the breakpoint is found (intron numbering is relative to the first translat ed exon). Genes completely deleted are listed in the final column. **Table S6 in File S2**: (a) Point mutations, indels, structural variations, and copy number variations presented in Figure 1. (b) TERT promoter mutations identified in 13 WGS cases: See separate.xlsx file. **Table S8 in File S2**: (a)Average haploid coverage across each targeted gene in the extension experiment. (b) Extension discovery variant table (15 paired samples): See separate.xlsx file. **Table S9 in File S2**: (a) Genes in the MAPK and Cell Cycle TP53/RB pathways. (b) Nonsynonymous mutations in the MAPK pathway in 28 paired discovery cases (c) Nonsynonymous mutations in the Cell Cycle TP53/RB pathway in 28 paired discovery cases: See separate.xlsx file. **Table S10 in File S2**: Extension analysis filtered variant table (96 unpaired samples): See separate.xlsx file. **Table S11 in File S2**: Extension analysis truncation mutation table (96 unpaired samples): See separate.xlsx file. **Table S12 in File S2**: (a)Readcounts for point mutations pictured in Figure 5. (b)Readcounts for point mutations pictured in Figure 6, MEL5 Lung sample. (c) Readcounts for point mutations pictured in Figure 6, MEL5 Pancreas sample. (d) Readcounts for point mutations pictured in Figure 6, MEL13 Lung sample. (e)Readcounts for point mutations pictured in Figure 6, MEL13 Chest Wall sample.(f) Readcounts for point mutations pictured in Figure 6, MEL5 sample.(g) Readcounts for point mutations pictured in Figure 6, MEL13 sample: See separate.xlsx file. **Table S14 in File S2**: Nonsynonymous mutations in the four metastases samples of MEL167 along with the readcounts and annotation: See separate.xlsx file.
(ZIP)
