# Supplementary material for: Clonal Architectures and Driver Mutations in Metastatic Melanomas
Source: PLoS One. 2014 Nov 13;9(11):e111153. doi: 10.1371/journal.pone.0111153 (PMC4230926; doi:10.1371/journal.pone.0111153)
Supplement: File S1 — Supplementary Materials and Methods, along with Supplementary Figures S1 to S4 and Supplementary Tables S1 to S3, S7, S13 and S15 and references cited in the Supplementary materials and method section. Figure S1 in File S1: Copy number patterns and structural variants identified in all 15 tumors sequenced. Figure S2 in File S1: Extension heatmap of recurrent BRAF and NRAS mutations as well as truncation mutations in selected tumors suppressor, protein phosphatase and chromatin remodeling genes. The mutual exclusion and co-occurrence of common recurrent drivers and truncation mutations are shown in the 96 extension cases. Three melanoma cases harbored both BRAF and NRAS mutations. Figure S3 in File S1: Density plots of variants to estimate the purity of the four metastases for each patient MEL167 and MEL174. Both the density plots and the Scatter plots were used for purity estimation of the metastases samples. Figure S4 in File S1: Flowchart showing the detailed overview of the analysis steps and the pipeline used for the analysis. Table S1 in File S1: WGS haploid coverage and SNP array concordance. Haploid and diploid coverage estimates are given for 15 whole-genome sequenced samples. Haploid coverage is calculated as the amount of non-redundant mapped read bases divided by the haploid size of the human genome. Diploid coverage is estimated from the fraction of heterozygous SNPs from high-density SNP array data that were present in SAMtools raw (unfiltered) or filtered SNP calls. Table S2 in File S1: Capture validation coverage. Custom capture validation coverage of putative somatic mutations is reported for the 13 cases in which such data were generated. Shown are the fraction of bases targeted that were covered >1x,>10x, and >20x in each sample. Table S3 in File S1: Tier 1–3 somatic SNVs predicted and validation rate. Numbers of validated somatic SNVs in tiers 1, 2, and 3 are shown for the 13 cases having both whole genome sequence data and custom capture validati [file pone.0111153.s001.docx]

# Clonal Architectures and Driver Mutations in Metastatic Melanomas

Li Ding^1,2,3,4*^, Minjung Kim^5*^, Krishna L. Kanchi^1*^, Nathan D. Dees^1^, Charles Lu^1^, Malachi Griffith^1^, David Fenstermacher^5^, Hyeran Sung^5^, Christopher A. Miller^1^, Brian Goetz^6^, Michael C. Wendl^1^, Obi Griffith^1,2^, Lynn A. Cornelius^2,6^, Gerald P. Linette^2,4^, Joshua F. McMichael^1^ , Vernon K Sondak^5^, Ryan C. Fields^4,6^, Timothy J. Ley^1,2,4^, James J. Mulé^5^, Richard K. Wilson^1,3,4^, and Jeffrey S. Weber^5^

^1^The Genome Institute, ^2^Department of Medicine, ^3^Department of Genetics, ^4^Siteman Cancer Center, ^6^Department of Surgery, Washington University in St. Louis, MO 63108, USA

^5^Donald A. Adam Comprehensive Melanoma Research Center, Moffitt Cancer Center, Tampa, FL 33612, USA

Address correspondence to:

Jeffrey Weber M.D. Ph.D.

Moffitt Cancer Center

12902 Magnolia Drive, SRB-2

Tampa, FL 33612

Jeffrey.weber@moffitt.org

*These authors contributed equally to this work.

**Table of Contents***

1. Supplementary Materials and Methods (pp. 3 - 8)
2. Supplementary Figures (pp. 9 - 13)
3. Supplementary Tables (pp. 15- 23)
4. References (pp. 24 - 25)

**A.Supplementary Materials and Methods**

**Capture array design**

We used custom sequence capture arrays from Roche Nimblegen to validate putative mutations. To perform capture validation for individual samples, (1) for cases with fewer than 10,000 total sites, we selected all predicted indels and SNVs from non-repetitive regions of the genome (tiers 1-3) as well as genome-wide SVs; (2) for cases with greater than 10,000 sites, we included all SVs, tier 1 SNVs, tier 1 indels, and based on the tier 2 and tier 3 SNVs counts, we randomly picked tiers 2 and 3 sites to reach 10,000 sites/genome. Nimblegen was able to design capture probes for between 81.1% and 99.9% of the intended targets. Solid phase captures were performed as previously described[^7^](#_ENREF_7).

Here are the SNVs counts that were sent to validation for each case.

MEL1: all tier 1, all tier 2, and all tier 3 sites were included for validation.

MEL2: all tier 1, all tier 2, and 5961 out of 17885 (33%) tier 3 sites were selected for validation.

MEL3: all tier 1, all tier 2, and 3417 out of 30761 (11%) tier 3 sites were selected for validation.

MEL4: all tier 1, 4013 out of 20065 (20%) tier 2 sites, and 2691 out of 107661 (2%) tier 3 sites were selected for validation.

MEL5: as we have two metastasis samples, the duplicates were removed and then all tier 1, 5147 out of 10294 (50%) tier 2 sites, and 2630 out of 55248 (5%) tier 3 sites were selected for validation.

MEL6: all tier 1, 4629 out of 23145 (20%) tier 2 sites, and 2734 out of 128524 (2%) tier 3 sites were selected for validation.

MEL7: all tier 1, 5309 out of 10618 (50%) tier 2 sites, and 2698 out of 56674 (4%) tier 3 sites were selected for validation.

MEL8: all tier 1, all tier 2, and 3067 out of 30672 (10%) tier 3 sites were selected for validation.

MEL9: all tier 1, 1473 out of 47157 (3%) tier 2 sites, and 986 out of 214122 (0.4%) tier 3 sites were selected for validation.

MEL10: all tier 1, all tier 2, and 5694 out of 20382 (28%) tier 3 sites were selected for validation.

MEL11: all tier 1, 4939 out of 9879 (50%) tier 2 sites, and 3103 out of 52761 (6%) tier 3 sites were selected for validation.

MEL12: all tier 1, all tier2, and 3216 out of 28944 (11%) tier 3 sites were selected for validation.

MEL13: as we have two metastasis samples, the duplicates were removed and then all tier 1, all tier2, and 4111 out of 24669 (17%) tier 3 sites were selected for validation.

For small insertions and SNVs, the targeted regions were exactly 200bp centered on the variant. For small deletions, the deleted sequence plus 100 bp of sequence flanking each end of the deletion were selected.

For putative somatic SVs, we requested probes tiled across the predicted breakpoint flanking 100bp of the outermost, predicted breakpoint. For larger insertions a single region was requested, but for translocations, deletions, and inversions etc, we requested two targets, one for each breakpoint. Roche Nimblegen design parameters allowed for probes with up to five additional sequence matches elsewhere in the genome.

**Alignment of solid phase capture validation data**

We generated 100bp paired-end sequence data using one Illumina HiSeq lane for each metastastatic tumor or normal sample. Illumina reads were mapped to the NCBI Build 36 reference sequence (BWA v0.5.9), merged into BAM files (SAMtools v1 r599), and duplicate reads were tagged (Picard v1.29). Coverage of target sequences was assessed using RefCov software (T. Wylie et al, unpublished). We obtained greater than 20X haploid reference coverage for 96.9 to 99.68% of the targeted sites in each sample pair (Supplementary Table 2).

**Validation of SNVs and dinucleotide variants**

Putative SNVs and dinucleotide variants were validated using VarScan 2 (http://varscan.sourceforge.net) with the following parameters:

*-min-coverage 30*

*-min-var-freq 0.08*

*-normal-purity 1*

*-p-value 0.10*

*-somatic-p-value 0.001*

*-validation 1*

Based on the allele frequency and reads supporting reference and variant alleles at the position of each predicted variant in the tumor and normal BAMs, VarScan classifies each putative somatic event as Reference (wildtype), Germline, Somatic, or LOH. Validated somatic mutations are further filtered with additional filters that removes false positives supported by strand specific artifacts, read position artifacts, or poorly mapped reads. Potentially ambiguous sites were further resolved with additional visualization of the primary and validation data.

**Validation of indels**

***Small (1-2 bp) Indel Validation with Solid-Phase Capture Validation Data***

Putative indels 1-2 bp in size were converted to BED format and provided as the target intervals for the GATK IndelRealigner algorithm. BAM files for the tumor and matched normal were re-aligned independently using this set of target intervals.

To validate the original predictions, we developed a matching algorithm that attempts to match VarScan validation calls with the original indel predictions. Specifically, the algorithm searched for a validated indel of same type (insertion or deletion) and similar size (within 1 bp). To allow for differences in gapped alignments, the algorithm allowed matches at slightly different genomic positions, so long as the validated indel mapped within a specified interval (indel_size + 2bp) of the original prediction. Matched indels reported “Somatic” in the tumor sample were manually reviewed in the re-aligned BAM files using IGV to visualize the data.

***Medium (3-100 bp) Indel Validation with Solid-Phase Capture Validation Data***

Sample validation data for indels 3-100bp, in size were assembled using the TIGRA assembler (Chen et al. unpublished). Breakpoints and microhomology were identified using Crossmatch alignments (version 1.080721, Green unpublished). We then sized the chosen contigs to 500 bp length by trimming excess sequence or padding from the reference sequence and compared overlapping contigs using the dpAlign module of BioPerl (http://www.bioperl.org/wiki/Main_Page) to generate an “Ends-free” alignment between the two pairs. If an alignment contained no gaps, shared at least 98% sequence identity and had a length of at least 95 bp then the leftmost contig that aligned to the reference was retained. Contigs that remained after merging were concatenated to the NCBI build36 reference sequence as additional novel contigs and the validation reads were mapped back to the expanded reference using BWA and depduplicated using Picard (http://sourceforge.net/apps/mediawiki/picard). Those with a mapping quality greater than 0 that completely spanned the established indel breakpoints without gaps in the alignment were identified. Variants with greater than 30 reads aligning to either the reference or the indel contig and a variant allele frequency difference of greater than 10% between any two samples were manually reviewed.

**Within-clone mutation spectrum analysis**

The control data for this analysis was created by querying 10,000 random cytosine base locations on chromosome 2 of the NCBI human build 36 reference sequence, and determining the proportion of cases in which the cytosine base was located at the 3’ end of a pyrimidine dinucleotide. This count was performed ten times, resulting in the expectation that this dipyrimidine scenario should occur at 54.57 ± 0.05% of all cytosine bases by mere chance. This is comparable to the expected value of 53% reported previously[^8^](#_ENREF_8).

SNVs which represented C->T transitions and whose variant allele frequencies were within 3% of the peak variant allele frequencies of each sub-clone in bi-clonal tumors MEL9 and MEL10 (**Figure 6**) were identified and queried in a similar manner for the proportion of C->T transitions with preceding pyrimidine bases. A standard proportion test was performed independently for each sub-clone to determine whether or not the rate of occurrence of diyrimidine bases at C->T transition sites in each subclone matched the expected value, or instead differed significantly from the expected value.

**Tumor sample selection**

The 15 tumors sequenced were chosen from a group of over 500 melanomas that are included in a clinically annotated frozen tumor bank maintained at the Moffitt Cancer Center, Tampa, FL. The specimens were all macro-dissected to contain at least 80% tumor and frozen within 30 minutes of surgical resection according to SOPs at the Moffitt Cancer Center. All samples were screened for the presence of metastatic melanoma based on surgical pathology reports and clinical records, and all medical records were reviewed and verified by one of the authors (JSW). DNA extracted from chosen tumor specimens and corresponding normal peripheral blood mononuclear cells (PBMC) were subjected to additional quality control measures. The 15 chosen tumors had matched normal PBMC available and had frozen tumor available for analysis. At least 2 or 3 tumors were chosen from different anatomic sites, and at least 2 patients were chosen to have metachronous metastases. The tumor and normal tissue were obtained under an Institutional Review Board (IRB) approved approval for the Moffitt Total Cancer Care initiative, and a separate IRB approved protocol was written for the sequencing phase.

**Extension analysis**

25 additional melanoma tumors from 15 paired-normal samples as well as 97 additional melanoma tumors (unpaired) from 96 patients were captured across 1,209 genes of interest as described above. All nonsilent events discovered in the 15 paired extension samples were reviewed manually for somatic status using IGV, and are reported in Supplemental Table 6b. From unpaired extension samples, we are reporting truncation events (nonsense SNVs, splice site SNVs, nonstop SNVs, and frame shift indels) in Supplemental Table 9, all truncation events in Supplemental Table 9 were reviewed manually using IGV. Events discovered in the unpaired extension samples were filtered by position to include those events found previously in either: our original 15 WGS samples, our 15 paired extension samples, recent melanoma literature [^10-14^](#_ENREF_10), or in the COSMIC database [^15^](#_ENREF_15). All filtered events from these unpaired tumor samples occuring in more than one patient were manually reviewed in IGV in the discovery tumor sample in order to eliminate alignment artifacts, as well as in two random melanoma normal samples from the paired extension dataset in order to eliminate obvious germline variants.

**Phylip Analysis of the Multi-metastases samples**

Nucleotide sequences from normal and metastatic genomes were compared phylogenetically using the PHYLIP package (CITE Felsenstein, J. 1989. PHYLIP - Phylogeny Inference Package (Version 3.2). Cladistics 5: 164-166) for the two data sets which had a sufficient number of members to generate trees, MEL 167 and 174. First, variant allele frequencies (VAFs) were estimated from digital read counts and purity estimates were made from density and scatter plots of the variants. We found purities for mets 1 through 4 to be approximately 30%, 75%, 75%, and 60%, respectively, in MEL 167 and 20%, 90%, 60%, and 30%, respectively, in MEL 174. These values were then used to correct VAFs and the resulting information was written to PHYLIP-formatted input files for the two data sets. We then used the PHYLIP tool "contml" to generate phylogenetic trees based upon the maximum likelihood approach, where the normal sample was specified as the out-group. Finally, PHYLIP's "drawtree" tool was used to render unrooted phylogenetic trees for the two data sets from their contml outputs.

**B. Supplementary Figures**

**Figure S1**


**2**

**Figure S2**

**
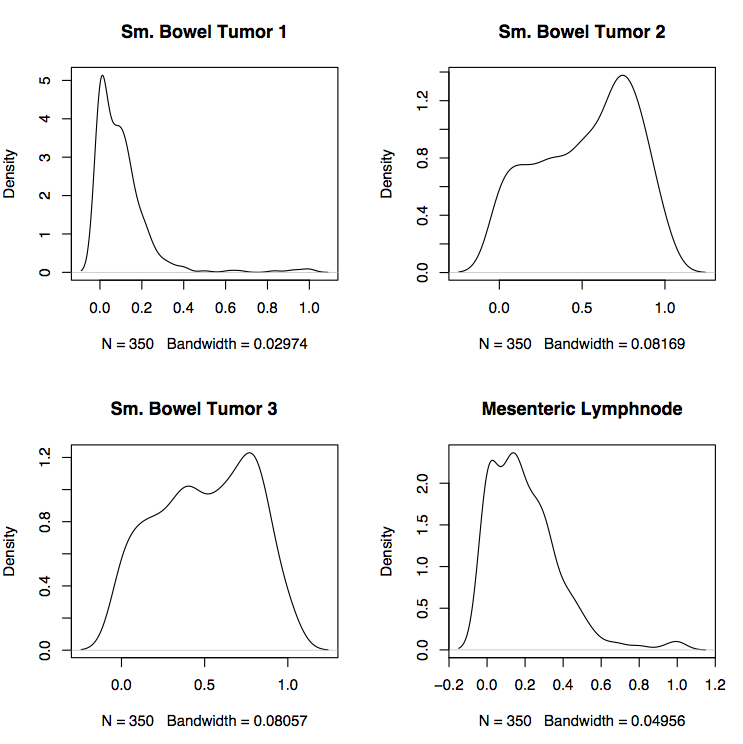
**

**Figure S3**

**H**

**MEL167**

**
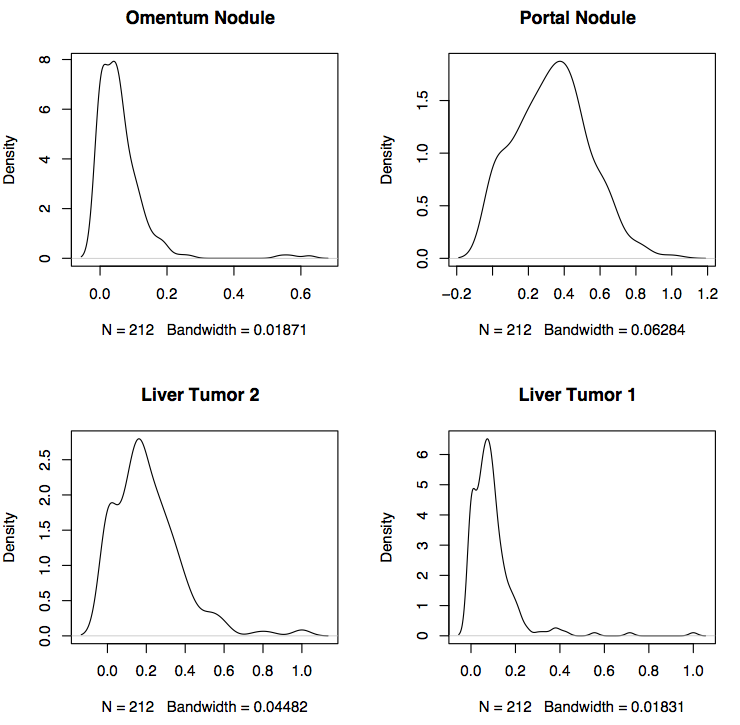
**

**MEL174**

**Figure S4**

**D. Supplementary Tables**

| **Sample** | **Sequenced Kilobases** | **Number of reads mapped** | **Heterozygous SNP concordance (%)** | **Haploid Coverage (%)** |
| --- | --- | --- | --- | --- |
| **MEL1_normal** | **1603688704** | **1529941142** | **99.53** | **45.17** |
| **MEL1_tumor** | **1179273958** | **1135281874** | **99.42** | **30.44** |
| **MEL2_normal** | **1188774748** | **1136769719** | **99.59** | **34.83** |
| **MEL2_tumor** | **1543037744** | **1486443116** | **99.36** | **38.73** |
| **MEL3_normal** | **1562782348** | **1497639566** | **99.57** | **45.3** |
| **MEL3_tumor** | **1546461154** | **1508153794** | **99.12** | **45.83** |
| **MEL4_normal** | **1504250486** | **1431974081** | **99.57** | **43.94** |
| **MEL4_tumor** | **1374071424** | **1320162406** | **99.34** | **39.89** |
| **MEL5_normal** | **2130640144** | **2038147275** | **99.64** | **53.68** |
| **MEL5-lung** | **3041579183** | **2963871853** | **99.59** | **63.22** |
| **MEL5_pancreas** | **1236646104** | **1198941100** | **99.26** | **34.52** |
| **MEL6_normal** | **1672390702** | **1597103469** | **99.57** | **47.12** |
| **MEL6_tumor** | **1101294294** | **1061667483** | **98.66** | **29.51** |
| **MEL7_normal** | **1440573084** | **1373622764** | **99.59** | **41.23** |
| **MEL7_tumor** | **1257299688** | **1217116335** | **99.23** | **34.28** |
| **MEL8_normal** | **2137405104** | **2003195639** | **99.65** | **55.25** |
| **MEL8_tumor** | **1443847726** | **1402261775** | **99.53** | **39.47** |
| **MEL9_normal** | **1220458164** | **1160503068** | **99.53** | **34.92** |
| **MEL9_tumor** | **1326195000** | **1280438156** | **99.39** | **31.32** |
| **MEL10_normal** | **1468609120** | **1401150429** | **99.54** | **41.89** |
| **MEL10_tumor** | **1248457498** | **1197373589** | **98.98** | **29.57** |
| **MEL11_normal** | **2030407080** | **1931866266** | **99.66** | **51.74** |
| **MEL11_tumor** | **1477205038** | **1433517523** | **99.4** | **44.03** |
| **MEL12_normal** | **1607215572** | **1532344131** | **99.51** | **45.97** |
| **MEL12_tumor** | **1158487418** | **1120144192** | **99.18** | **31.78** |
| **MEL13_normal** | **1274716936** | **1214549836** | **99.59** | **36.58** |
| **MEL13_lung** | **1330258070** | **1277215114** | **99.1** | **39.08** |
| **MEL13_chestwall** | **1302218588** | **1222704620** | **98.94** | **37.48** |

**Table S1 in File S1: WGS haploid coverage and SNP array concordance**

Haploid and diploid coverage estimates are given for 15 whole-genome sequenced samples. Haploid coverage is calculated as the amount of non-redundant mapped read bases divided by the haploid size of the human genome. Diploid coverage is estimated from the fraction of heterozygous SNPs from high-density SNP array data that were present in SAMtools raw (unfiltered) or filtered SNP calls.

| ID | Sequenced Kilobases | Number of reads mapped | 1X | 10X | 20X |
| --- | --- | --- | --- | --- | --- |
| MEL1_normal | 222817994 | 216543254 | 99.81% | 99.45% | 99.24% |
| MEL1_tumor | 189792404 | 187485962 | 99.82% | 99.48% | 99.27% |
| MEL2_normal | 197575496 | 191062613 | 99.91% | 99.76% | 99.67% |
| MEL2_tumor | 192492320 | 190512185 | 99.83% | 99.63% | 99.53% |
| MEL3_normal | 188013540 | 180562425 | 99.86% | 99.65% | 99.52% |
| MEL3_tumor | 202554718 | 200304107 | 99.86% | 99.66% | 99.56% |
| MEL4_normal | 202319298 | 193385684 | 98.33% | 97.32% | 96.99% |
| MEL4_tumor | 163024948 | 157808514 | 97.20% | 89.07% | 79.11% |
| MEL5_normal | 195837016 | 192838966 | 99.71% | 99.21% | 98.97% |
| MEL5_lung | 209559688 | 207259474 | 99.72% | 99.23% | 98.96% |
| MEL5_pancreas | 197383226 | 195328290 | 99.76% | 99.31% | 99.07% |
| MEL6_normal | 184003498 | 181437545 | 99.40% | 99.06% | 98.93% |
| MEL6_tumor | 205014304 | 202675647 | 99.37% | 99.00% | 98.85% |
| MEL7_normal | 231809888 | 228243468 | 98.75% | 97.94% | 97.71% |
| MEL7_tumor | 188876828 | 186599231 | 98.68% | 97.91% | 97.62% |
| MEL8_normal | 170846694 | 170846694 | 99.23% | 98.66% | 98.48% |
| MEL8_tumor | 161889810 | 160201516 | 99.14% | 98.71% | 98.51% |
| MEL9_normal | 210308192 | 205052444 | 99.90% | 99.69% | 99.56% |
| MEL9_tumor | 200984996 | 195326067 | 99.89% | 99.71% | 99.58% |
| MEL10_normal | 215491904 | 211950533 | 99.89% | 99.76% | 99.68% |
| MEL10_tumor | 197810594 | 193763078 | 99.90% | 99.74% | 99.64% |
| MEL11_normal | 208747634 | 205215862 | 99.65% | 99.25% | 99.05% |
| MEL11_tumor | 221767680 | 214454268 | 99.60% | 99.16% | 98.95% |
| MEL12_normal | 214249568 | 209944318 | 99.85% | 99.47% | 99.22% |
| MEL12_tumor | 228854384 | 225160943 | 99.81% | 99.32% | 99.04% |
| MEL13_normal | 222104780 | 219044365 | 99.58% | 98.97% | 98.65% |
| MEL13_lung | 197993046 | 193498289 | 99.51% | 98.79% | 98.44% |
| MEL13_chestwall | 214552056 | 211913775 | 99.60% | 99.06% | 98.77% |

**Table S2 in File S1: Capture validation coverage**

Custom capture validation coverage of putative somatic mutations is reported for the 13 cases in which such data were generated. Shown are the fraction of bases targeted that were covered >1x, >10x, and >20x in each sample.

| **Sample** | **T123 predicted SNVs** | **T123 SNVs sent to Validation** | **T123 SNVs Validated passed filters** | **% Validated after filters** |
| --- | --- | --- | --- | --- |
| MEL1 | 7209 | 7202 | 6580 | 91.36 |
| MEL2 | 21570 | 9638 | 9441 | 97.96 |
| MEL3 | 36967 | 9617 | 9007 | 93.66 |
| MEL4 | 130014 | 8990 | 5918 | 65.83 |
| MEL5 | 66708 | 8940 | 8719 | 97.53 |
| MEL6 | 153934 | 9623 | 9244 | 96.06 |
| MEL7 | 68381 | 9094 | 8751 | 96.23 |
| MEL8 | 36908 | 9297 | 8858 | 95.28 |
| MEL9 | 268516 | 9690 | 9077 | 93.67 |
| MEL10 | 24510 | 9817 | 8914 | 90.80 |
| MEL11 | 63872 | 9267 | 9071 | 97.88 |
| MEL12 | 34745 | 9001 | 8719 | 96.87 |
| MEL13 | 29629 | 9059 | 8777 | 96.89 |

**Table S3 in File S1: Tier 1-3 somatic SNVs predicted and validation rate**

Numbers of validated somatic SNVs in tiers 1, 2, and 3 are shown for the 13 cases having both whole genome sequence data and custom capture validation.

| **Sample** | **T123 DNP** | **T123 SNVs unvalidated** | **% of T123DNPs/SNV unvalidated** | **Tier1 DNP** | **Tier1 SNVs** | **% of T1 DNPs /SNV** | **%Tier1DNP/T123DNP** | **CC-TT** |
| --- | --- | --- | --- | --- | --- | --- | --- | --- |
| MEL1 | 93 | 7209 | 1.29 | 1 | 131 | 0.76 | 1.08 | 68 |
| MEL2 | 361 | 21570 | 1.67 | 16 | 373 | 4.29 | 4.43 | 73 |
| MEL3 | 644 | 36967 | 1.74 | 28 | 607 | 4.61 | 4.35 | 73 |
| MEL4 | 2171 | 130014 | 1.67 | 76 | 1781 | 4.27 | 3.50 | 74 |
| MEL5l | 687 | 50831 | 1.35 | 14 | 1023 | 1.37 | 2.04 | 76 |
| MEL5p | 886 | 64984 | 1.36 | 17 | 1136 | 1.50 | 1.92 | 75 |
| MEL6 | 2291 | 153934 | 1.49 | 66 | 2201 | 3.00 | 2.88 | 72 |
| MEL7 | 1029 | 68381 | 1.50 | 39 | 1056 | 3.69 | 3.79 | 77 |
| MEL8 | 530 | 36908 | 1.44 | 19 | 614 | 3.09 | 3.58 | 73 |
| MEL9 | 1245 | 268515 | 0.46 | 38 | 1781 | 2.13 | 3.05 | 76 |
| MEL10 | 423 | 24510 | 1.73 | 15 | 404 | 3.71 | 3.55 | 75 |
| MEL11 | 1104 | 63872 | 1.73 | 33 | 1207 | 2.73 | 2.99 | 76 |
| MEL12 | 845 | 34745 | 2.43 | 34 | 541 | 6.28 | 4.02 | 78 |
| MEL13c | 653 | 28299 | 2.31 | 25 | 497 | 5.03 | 3.83 | 75 |
| MEL13l | 592 | 27097 | 2.18 | 22 | 500 | 4.40 | 3.72 | 75 |

**Table S7 in File S1: Dinucleotide polymorphisms (DNP) in 13 WGS cases.**

| **Sample** | **Metastases** | **Purity estimation** |
| --- | --- | --- |
| MEL167 | Small bowel tumor1 | 30.00% |
|  | Small bowel tumor2 | 75.00% |
|  | Small bowel tumor3 | 75.00% |
|  | Mesentric Lymphnode | 60.00% |
|  |  |  |
| MEL174 | Liver tumor1 | 30.00% |
|  | Liver tumor2 | 60.00% |
|  | Portal nodule | 90.00% |
|  | Omentum | 20.00% |

**Table S13 in File S1: Purity estimation of the multi-metastases samples MEL167 and MEL174 using the density plots**

| Genes | Number of samples in WGS | Number of samples in TCGA | Percent of samples in WGS | Percent of samples in TCGA |
| --- | --- | --- | --- | --- |
| ATM | 1 | 22 | 6.67 | 8.70 |
| BRAF | 13 | 140 | 86.67 | 55.34 |
| CDK2 | 2 | 0 | 13.33 | 0.00 |
| CDKN1A | 1 | 5 | 6.67 | 1.98 |
| CDKN2A | 2 | 34 | 13.33 | 13.44 |
| EGFR | 2 | 39 | 13.33 | 15.42 |
| EPHA3 | 10 | 35 | 66.67 | 13.83 |
| EPHA7 | 8 | 56 | 53.33 | 22.13 |
| FGFR1 | 2 | 15 | 13.33 | 5.93 |
| FGFR4 | 2 | 16 | 13.33 | 6.32 |
| GRIN2A | 9 | 123 | 60.00 | 48.62 |
| GRIN2B | 7 | 81 | 46.67 | 32.02 |
| GRIN3A | 4 | 42 | 26.67 | 16.60 |
| GRM1 | 3 | 23 | 20.00 | 9.09 |
| GRM3 | 5 | 74 | 33.33 | 29.25 |
| GRM6 | 1 | 33 | 6.67 | 13.04 |
| GRM7 | 3 | 55 | 20.00 | 21.74 |
| GRM8 | 3 | 68 | 20.00 | 26.88 |
| HNF4G | 6 | 29 | 40.00 | 11.46 |
| JAK1 | 1 | 7 | 6.67 | 2.77 |
| JAK3 | 1 | 11 | 6.67 | 4.35 |
| KIT | 2 | 12 | 13.33 | 4.74 |
| MAP2K4 | 3 | 4 | 20.00 | 1.58 |
| MAP2K5 | 3 | 3 | 20.00 | 1.19 |
| MAP3K1 | 2 | 6 | 13.33 | 2.37 |
| MAP3K14 | 2 | 7 | 13.33 | 2.77 |
| MAP3K4 | 2 | 18 | 13.33 | 7.11 |
| MAP3K5 | 1 | 23 | 6.67 | 9.09 |
| MAP3K7 | 1 | 4 | 6.67 | 1.58 |
| MAP3K9 | 1 | 32 | 6.67 | 12.65 |
| MAP4K2 | 1 | 6 | 6.67 | 2.37 |
| MAPK10 | 3 | 10 | 20.00 | 3.95 |
| MAPK4 | 1 | 28 | 6.67 | 11.07 |
| MAPK7 | 2 | 5 | 13.33 | 1.98 |
| MDM2 | 2 | 4 | 13.33 | 1.58 |
| NRAS | 3 | 71 | 20.00 | 28.06 |
| PDGFRB | 3 | 23 | 20.00 | 9.09 |
| PIK3C2A | 2 | 12 | 13.33 | 4.74 |
| PIK3C2B | 5 | 27 | 33.33 | 10.67 |
| PIK3C2G | 6 | 56 | 40.00 | 22.13 |
| PIK3C3 | 2 | 4 | 13.33 | 1.58 |
| PIK3CA | 2 | 8 | 13.33 | 3.16 |
| PIK3CG | 3 | 27 | 20.00 | 10.67 |
| PIK3R5 | 5 | 20 | 33.33 | 7.91 |
| PIK3R6 | 3 | 11 | 20.00 | 4.35 |
| RB1 | 1 | 11 | 6.67 | 4.35 |
| STAT1 | 1 | 6 | 6.67 | 2.37 |
| STAT5A | 2 | 3 | 13.33 | 1.19 |
| STAT6 | 1 | 10 | 6.67 | 3.95 |
| TP53 | 1 | 41 | 6.67 | 16.21 |
| ZNF831 | 9 | 104 | 60.00 | 41.11 |
| ASXL3 | 9 | 94 | 60.00 | 37.15 |
| PTPRT | 13 | 157 | 86.67 | 62.06 |

**Table S15 in FileS1: Comparison of the number of Tier1 SNVs in the TCGA melanoma dataset to the number of SNVs in the WGS dataset.**

1. **References**

1. Mardis, E.R. *et al.* Recurring mutations found by sequencing an acute myeloid leukemia genome. *N Engl J Med* **361**, 1058-66 (2009).

2. Li, H. *et al.* The Sequence Alignment/Map format and SAMtools. *Bioinformatics* **25**, 2078-9 (2009).

3. Larson, D.E. *et al.* SomaticSniper: Identification of Somatic Point Mutations in Whole Genome Sequencing Data. *Bioinformatics* (2011).

4. Ye, K., Schulz, M.H., Long, Q., Apweiler, R. & Ning, Z. Pindel: a pattern growth approach to detect break points of large deletions and medium sized insertions from paired-end short reads. *Bioinformatics* **25**, 2865-71 (2009).

5. McKenna, A. *et al.* The Genome Analysis Toolkit: a MapReduce framework for analyzing next-generation DNA sequencing data. *Genome research* **20**, 1297-303 (2010).

6. Chen, K. *et al.* BreakDancer: an algorithm for high-resolution mapping of genomic structural variation. *Nat Methods* **6**, 677-81 (2009).

7. Ding, L. *et al.* Clonal evolution in relapsed acute myeloid leukaemia revealed by whole-genome sequencing. *Nature* **481**, 506-10 (2012).

8. Pleasance, E.D. *et al.* A comprehensive catalogue of somatic mutations from a human cancer genome. *Nature* **463**, 191-6 (2010).

9. Dees, N.D. *et al.* MuSiC: Identifying mutational significance in cancer genomes. *Genome research* **22**, 1589-98 (2012).

10. Stark, M.S. *et al.* Frequent somatic mutations in MAP3K5 and MAP3K9 in metastatic melanoma identified by exome sequencing. *Nat Genet* **44**, 165-9 (2011).

11. Wei, X. *et al.* Exome sequencing identifies GRIN2A as frequently mutated in melanoma. *Nat Genet* **43**, 442-6 (2011).

12. Hodis, E. *et al.* A landscape of driver mutations in melanoma. *Cell* **150**, 251-63 (2012).

13. Prickett, T.D. *et al.* Analysis of the tyrosine kinome in melanoma reveals recurrent mutations in ERBB4. *Nat Genet* **41**, 1127-32 (2009).

14. Prickett, T.D. *et al.* Exon capture analysis of G protein-coupled receptors identifies activating mutations in GRM3 in melanoma. *Nat Genet* **43**, 1119-26 (2011).

15. Forbes, S.A. *et al.* The Catalogue of Somatic Mutations in Cancer (COSMIC). *Curr Protoc Hum Genet* **Chapter 10**, Unit 10 11 (2008).

16. Serrano, M. *et al.* Role of the INK4a locus in tumor suppression and cell mortality. *Cell* **85**, 27-37 (1996).

17. Sviderskaya, E.V. *et al.* Complementation of hypopigmentation in p-mutant (pink-eyed dilution) mouse melanocytes by normal human P cDNA, and defective complementation by OCA2 mutant sequences. *J Invest Dermatol* **108**, 30-4 (1997).

18. Nogueira, C. *et al.* Cooperative interactions of PTEN deficiency and RAS activation in melanoma metastasis. *Oncogene* **29**, 6222-32 (2010).
